# Epidemiology of herpes simplex virus type 2 in Asia: A systematic review, meta-analysis, and meta-regression

**DOI:** 10.1016/j.lanwpc.2021.100176

**Published:** 2021-06-09

**Authors:** Sawsan AlMukdad, Manale Harfouche, Anja Wettstein, Laith J. Abu-Raddad

**Affiliations:** a Infectious Disease Epidemiology Group, Weill Cornell Medicine-Qatar, Cornell University, Qatar Foundation - Education City, Doha, Qatar; b World Health Organization Collaborating Centre for Disease Epidemiology Analytics on HIV/AIDS, Sexually Transmitted Infections, and Viral Hepatitis, Weill Cornell Medicine–Qatar, Cornell University, Qatar Foundation – Education City, Doha, Qatar; c Department of Population Health Sciences, Weill Cornell Medicine, Cornell University, New York, New York, USA

**Keywords:** Prevalence, Incidence, Genital ulcer disease, Genital herpes, Synthesis, Region

## Abstract

**Background:**

Herpes simplex virus type 2 (HSV-2) infection is a prevalent sexually transmitted infection worldwide. This systematic review was conducted to characterize HSV-2 epidemiology in Asia, including the World Health Organization regions of Southeast Asia and the Western Pacific.

**Methods:**

Cochrane and PRISMA guidelines were followed to systematically review and report findings. Pooled mean seroprevalence and proportions of HSV-2 isolated in genital ulcer disease (GUD) and in genital herpes were calculated using random-effects meta-analyses. Meta-regressions were also conducted. Quality assessment was performed.

**Findings:**

HSV-2 measures extracted from 173 publications included 15 seroconversion rates, 11 seroincidence rates, 272 overall seroprevalence measures (678 stratified), 14 proportions of HSV-2 isolation in GUD (15 stratified), and 27 proportions of HSV-2 isolation in genital herpes (36 stratified). Pooled mean seroprevalence was 12.1% (95% confidence interval (CI): 11.0-13.2%) among general populations, 23.6% (95% CI: 20.9-26.3%) among men who have sex with men and transgender people, 46.0% (95% CI: 39.2-52.9%) among HIV-positive individuals and individuals in HIV-discordant couples, and 62.2% (95% CI: 58.9-65.6%) among female sex workers. Among general populations, pooled mean seroprevalence increased gradually from 4.7% (95% CI: 3.3-6.3%) in <20-year-old individuals to 26.6% (95% CI: 19.2-34.7%) in >60-year-old individuals. Compared to women and across all populations, men had 0.60-fold (95% CI: 54.0-67.0) lower seroprevalence, that is women had 70% higher seroprevalence. Seroprevalence declined by 0.98-fold (95% CI: 0.97-0.99) per year, that is a 2% decline per year in the last three decades. Pooled mean proportions of HSV-2 isolation in GUD and in genital herpes were 48.2% (95% CI: 34.9-61.6%) and 75.9% (95% CI: 68.3-82.8%), respectively.

**Interpretation:**

Over 1 in 10 individuals is infected with HSV-2, but seroprevalence is declining. HSV-2 accounts for half of GUD cases and three-quarters of genital herpes cases. These findings support the need for an HSV-2 vaccine and universal access to sexual and reproductive health services.

**Funding:**

This work was supported by the Qatar National Research Fund [NPRP 9-040-3-008] and by pilot funding from the Biomedical Research Program at Weill Cornell Medicine in Qatar.

**Research in context**

**Evidence prior to this study**

Herpes simplex virus type 2 (HSV-2) infection is a common sexually transmitted infection globally and causes a range of mild to severe disease outcomes. Despite decades of research on this infection, its global epidemiology remains insufficiently characterized. A PubMed search using a broad search strategy ("Herpes Simplex"[MeSH] AND "Review" [Publication Type]) identified no systematic review assessing HSV-2 epidemiology in Asia.

**Added value of this study**

Applying state-of-the-art methodologies, this study assessed HSV-2 epidemiology in Asia. We searched international electronic databases and found a large body of relevant epidemiological evidence that enabled detailed analyses and meta-analytics. We identified epidemiological associations and infection patterns of relevance in Asia and beyond. Robust and current estimates for HSV-2 seroincidence, seroprevalence, and proportions of HSV-2 isolation in genital ulcer disease (GUD) and in genital herpes were determined. Over 10% of Asians are infected, but seroprevalence is declining by 2% per year. Level of sexual risk behaviour, age, geography, and average national income explain much of the patterns of infection. HSV-2 infection was confirmed as the causal agent for 75% of genital herpes cases and 50% of GUD cases.

**Implications of available evidence**

In context of no public health programs targeted to prevent and control HSV-2 transmission, this infection was widespread, with serious disease outcomes. The scale of the disease burden requires prevention and control interventions that ensure universal access to sexual and reproductive healthcare services. With the infection's adverse consequences on reproductive, sexual, and psychosocial health, there is an urgent need to accelerate development of both prophylactic and therapeutic HSV-2 vaccines. Findings of the present study support the public health value and economic argument for HSV-2 vaccines as the fundamental solution to this global epidemic.

Alt-text: Unlabelled box

## Introduction

Herpes simplex virus type 2 (HSV-2) infection is a prevalent sexually transmitted infection (STI) worldwide.^1^^,^^2^ Although it is usually asymptomatic,[3], [4], [5] its chronic nature, with persistent reactivations and subclinical shedding, increases its transmission potential, resulting in higher prevalence than other STIs in both the general and higher-risk populations.[6], [7], [8], [9], [10]

HSV-2 infection manifests in the form of painful, itchy, and often recurrent ulcers in the genital tracts.[3], [4], [5]^,^^11^ It can also be transmitted vertically from mother to child, causing neonatal herpes, a disease condition associated with high morbidity and mortality.^12^^,^^13^ HSV-2 infection has been linked to a nearly 3-fold increase in HIV acquisition and transmission,[14], [15], [16] indicating an epidemiological synergy between the two infections.[17], [18], [19] Due to the high burden of disease caused by HSV-2, the World Health Organization (WHO) and global partners are leading efforts to develop an HSV-2 vaccine as an urgent priority.^20^^,^^21^

STI control has long languished on health policy agendas. To address this situation, the WHO formulated the “Global Health Sector Strategy on STIs”,^22^ adopted in 2016 by the 69^th^ World Health Assembly to guide the health-sector response to achieve the Sustainable Development Goals (SDGs).^23^ The strategy aims to eliminate STIs as a public health issue by 2030 through scale-up or incorporation of new prevention, control, and treatment interventions,^22^ as well as securing universal access to sexual and reproductive healthcare services.^22^^,^^23^ The first strategic direction of the strategy is “to understand the STI epidemic as a basis for advocacy, political commitment, national planning, resource mobilization and allocation, implementation, and program improvement.”^22^

In light of this strategy, this study comprehensively characterized HSV-2 epidemiology in Asia, including the WHO regions of Southeast Asia and the Western Pacific, through a systematic review of literature published during the last three decades. HSV-2 antibody prevalence (seroprevalence), HSV-2 seroincidence, proportion of HSV-2 isolation in genital ulcer disease (GUD), and proportion of HSV-2 isolation in genital herpes were investigated. Meta-analyses were conducted to estimate pooled means for HSV-2 seroprevalence, proportion of HSV-2 isolated in GUD, and proportion of HSV-2 isolated in genital herpes across various populations and subpopulations. Additionally, meta-regression analyses were performed to assess temporal trends in infection and to identify associations with higher seroprevalence.

## Methods

Methodology of this study was adapted from our published systematic reviews characterizing epidemiology of HSV-2 in Africa^24^ and epidemiology of HSV-1 in Asia^25^; therefore, no study protocol was registered in PROSPERO.

### Data sources and search strategy

The systematic literature review was guided by the Cochrane Collaboration Handbook.^26^ The reporting of findings followed the Preferred Reporting Items for Systematic Reviews and Meta-analyses (PRISMA) guidelines^27^ (Table S1).

The systematic literature search was performed using PubMed and Embase databases, until June 22, 2020, to identify relevant articles. MeSH/Emtree terms, keywords, and broad search criteria were used with no year or language restrictions to expand the search scope and to ensure inclusivity (Table S2). The Asian region as designated in this study, included 26 countries and was based on WHO's combined definitions for the two Asian regions of Southeast Asia and the Western Pacific.^28^ The 26 countries and their subregional classifications are listed in Box S1. Papua New Guinea was included as subregion of its own because of the uniqueness of this nation and for epidemiological relevance.

### Study selection and eligibility criteria

Two reviewers [BO (note dedication) and MH] independently screened retrieved articles. Search findings were imported into Endnote (Thomson Reuters, USA), where duplicate records were detected and deleted. Titles and abstracts of the remaining articles were screened for potential relevance, followed by full-text evaluation. Bibliographic screening of relevant articles and reviews was also conducted. Experts were consulted for additional data in the form of national public health reports.

In order to be eligible for inclusion in the study, publications had to report primary data in at least 10 subjects for any of the following four outcomes: (1) HSV-2 seroincidence, whether defined as the occurrence of infections per person-time or as a cumulative risk over a specific duration, (2) HSV-2 seroprevalence defined as the proportion of individuals tested that were seropositive, (3) the proportion of GUD cases in which HSV-2 was isolated as the cause of GUD, and (4) the proportion of genital herpes cases in which HSV-2 was isolated as the cause of the genital herpes.

Case reports, series, commentaries, reviews, and qualitative studies were excluded. In this study, “publication” refers to a document reporting any outcome measure, while a “study” refers to details of a specific outcome measure. Duplicate or overlapping studies were included only once.

### Data extraction and synthesis

Three reviewers (SM, AW, and MH) independently extracted and double-extracted data from included articles. Extracted variables are presented in Box S2.

Overall outcomes and their stratified measures were extracted based on a pre-set stratification hierarchy. Stratification for seroincidence and seroprevalence measures prioritized population type (defined in Box S3) followed by sex and age. Population type was determined based on the overall population type (Box S3), not factoring subpopulation overlaps of risk factors/behaviours. Stratification for the proportion of HSV-2 isolated in GUD or genital herpes prioritized genital herpes episode status (primary *versus* recurrent episodes) followed by sex and age, but there were too few studies with such stratifications to warrant further analysis. Outcome measures (seroincidence, seroprevalence, proportion of HSV-2 isolation in GUD, and proportion of HSV-2 isolation in genital herpes), if any, among subjects <15 years of age were only reported and were not included in analyses.

### Quality assessment

Due to recognized limitations related to sensitivity and specificity of HSV-2 diagnostic assays,^29^^,^^30^ a preliminary quality assessment was performed with the support of a leading expert in HSV-2 serological assays, Professor Rhoda Ashley-Morrow (University of Washington). Only studies that utilized valid and reliable assays were included. Precision of included studies and risk of bias (ROB) in included studies were assessed independently by three authors (SM, AW, and MH) following the Cochrane approach.^26^ Study precision was categorized as low *versus* high based on the overall sample size of the study (<200 *versus* ≥200), which was judged an acceptable level of precision for the level of seroprevalence observed in this study.^31^^,^^32^ Two quality domains were used to specify low *versus* high ROB: sampling method (probability-based *versus* non-probability-based) and response rate (≥80% *versus* <80% or unclear). If a study was based on preexisting medical records, the response rate was deemed unclear.

### Meta-analyses

The DerSimonian-Laird random-effects model^33^ was used for the meta-analysis. With the pooling of proportions, the Freeman-Tukey double arcsine transformation was applied in this analysis, using the command sm="PFT" in R,^34^ after ensuring applicability of this transformation.^35^ Pooled mean estimates for HSV-2 seroprevalence and pooled proportion of HSV-2 isolation in GUD and in genital herpes were calculated, by population and subpopulation, as long as each stratum had ≥3 measures.

Cochran's Q statistic was used to assess the presence of heterogeneity in effect size. I^2^ was used to measure the magnitude of between-study heterogeneity due to *true* differences in effect size instead of chance.^33^^,^^36^ Prediction interval, defined as the 95% interval of the distribution of true HSV-2 seroprevalence around the estimated mean, was used to describe the distribution of true effect sizes for each type of outcome.^33^^,^^36^ Meta-analyses were conducted in R, version 3.4.1 using the meta package.

### Meta-regressions

Using log-transformed seroprevalence measures, univariable and multivariable random-effects meta-regression analyses were conducted to investigate factors associated with increased HSV-2 seroprevalence and to explain inter-study heterogeneity. These factors were set *a priori* (Box S4).

All variables in the univariable analysis with a p-value<0.1 were included in multivariable models. A p-value<0.05 in the multivariable analysis indicated strong evidence for an association. Meta-regressions were conducted in Stata/SE version 13 ^37^ using the metareg package.^38^

### Role of the funding source

The funder of the study had no role in study design, data collection, data analysis, data interpretation, or writing of the article. The corresponding author had full access to all the data in the study and had the final responsibility for the decision to submit for publication.

## Results

### Search results and scope of evidence

**Figure 1:**
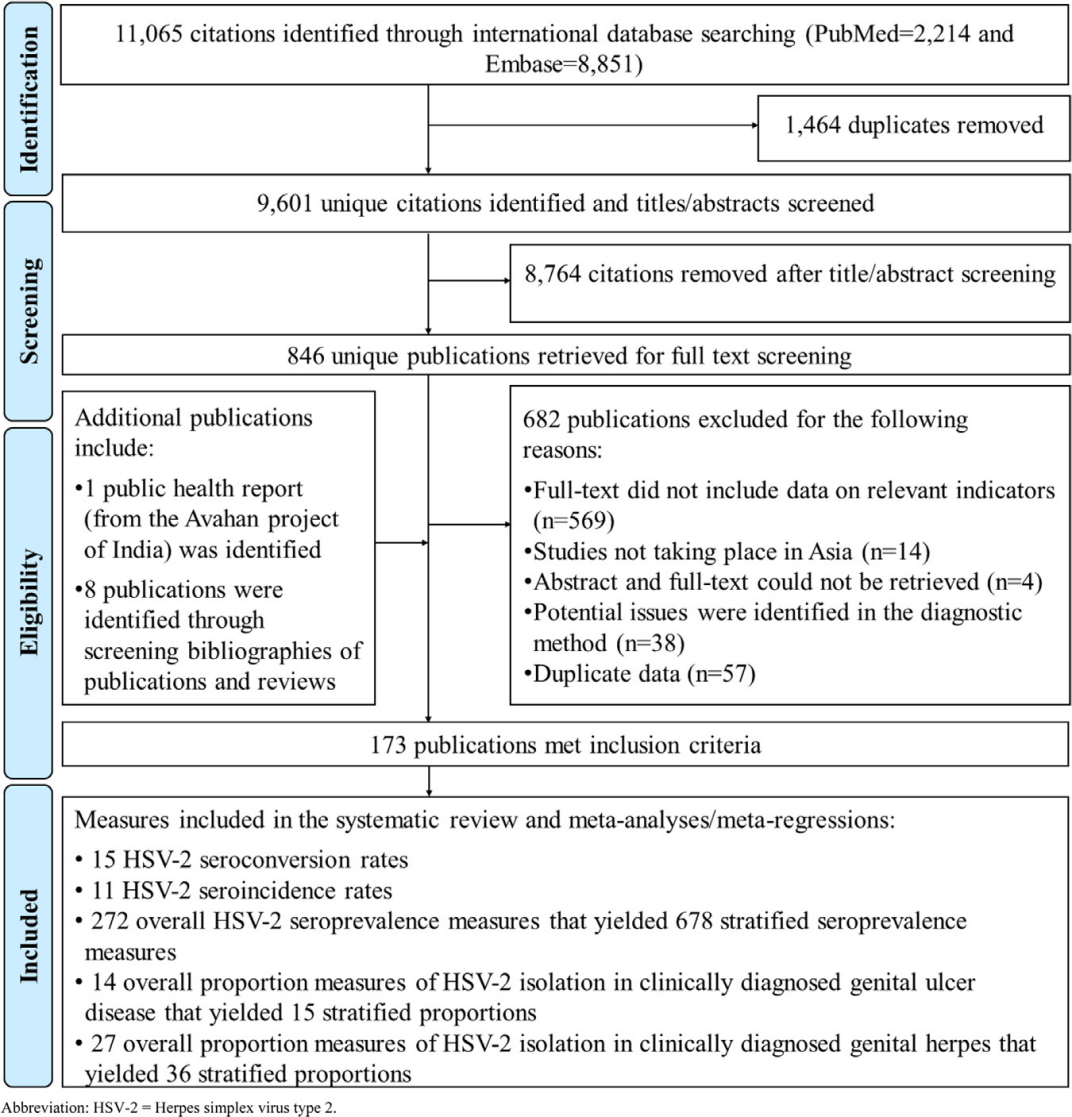
Flow chart of article selection for systematic review of HSV-2 infection in Asia, per PRISMA guidelines.^27^ Abbreviation: HSV-2 = Herpes simplex virus type 2.

A total of 11,065 articles (PubMed: 2,214 and Embase: 8,851) were retrieved and screened to identify eligible publications (Figure 1). One public health report was additionally provided by the Bill & Melinda Gates Foundation's Avahan project in India. Eight additional publications were identified through bibliographic screening of relevant articles.

Overall, 173 publications met the inclusion criteria. Extracted HSV-2 measures included 15 seroconversion rates, 11 seroincidence rates, 272 overall (that is based on the total sample of the study rather than specific stratum) seroprevalence measures (678 stratified measures), 14 overall proportions of HSV-2 isolation in GUD (15 stratified proportions), and 27 overall proportions of HSV-2 isolation in genital herpes (36 stratified proportions).

### Seroincidence overview

Seroconversion and seroincidence rates are reported in Table S3. Studies were mainly longitudinal cohorts (number of measures (n)=14; 93.3%) with a follow-up duration ranging from 6 months to 5.6 years. Among all population types, HSV-2 seroconversion rate and seroincidence rate ranged between 1.6-24.8% and 0.9-21.9 per 100 person-years, respectively, with the highest measures reported in a study of female sex workers (FSWs) in China.

### Seroprevalence overview and pooled mean estimates for HSV-2 seroprevalence

The majority of studies reporting seroprevalence measures (n=272) were published after 2005 (n=200; 73.5%) (Tables S4-S7). Most used convenience sampling (n=178; 65.4%). Stratified seroprevalence measures are summarized in Table 1, including ranges and medians by population type.

**Table 1:** Pooled mean estimates for HSV-2 seroprevalence in Asia.

Population type	Outcome measures	Samples	HSV-2 seroprevalence (%)		Pooled mean HSV-2 seroprevalence	Heterogeneity measures		
	Total n	Total N	Range	Median	Mean (%) (95% CI)	Q^a^ (p-value)	I²^b^ (%) (95% CI)	Prediction interval^c^ (%)
General populations	295	104,188	0.0-83.3	10.2	12.1 (11.0-13.2)	7,265.2 (p<0.001)	96.0 (95.7-96.2)	0.5-33.6
Women	169	71,983	0.0-71.0	11.7	13.3 (11.9-14.8)	4,568.9 (p<0.001)	96.3 (96.0-96.6)	1.1-34.6
Men	112	27,303	0.0-42.1	7.4	8.8 (7.4-10.3)	1,568.9 (p<0.001)	92.9 (92.0-93.8)	0.0-26.8
Mixed sexes	14	4,902	0.0-83.3	19.5	25.0 (17.4-33.5)	522.3 (p<0.001)	97.5 (96.8-98.1)	1.5-62.6
Intermediate-risk populations	86	23,946	2.0-80.4	21.0	20.2 (17.4-23.1)	2,178.6 (p<0.001)	96.1 (95.6-96.5)	2.1-49.2
Women	10	4,376	8.2 -72.7	18.9	24.8 (12.2-40.0)	748.6 (p<0.001)	98.8 (98.5-99.1)	0.0-82.7
Men	76	19,570	2.0-80.4	21.0	19.6 (17.0-22.4)	1,394.9 (p<0.001)	94.6 (93.8-95.3)	3.0-44.9
Higher-risk populations	209	73,139	3.4-100.0	50.0	47.9 (44.2-51.6)	19,834.7 (p<0.001)	99.0 (98.9-99.0)	5.5-92.3
FSWs	128	35,281	5.0-100.0	63.0	62.2 (58.9-65.6)	4,976.0 (p<0.001)	97.4 (97.2-97.7)	25.6-92.4
MSM, MSWs, and transgender people	79	37,554	3.4-78.7	22.5	23.6 (20.9-26.3)	2,664.9 (p<0.001)	97.1 (96.7-97.4)	5.3-49.2
Mixed sexes	2^d^	304	78.2-92.9	85.5	85.8 (69.1-96.9)	-	-	-
STI clinic attendees and symptomatic populations	46	6,610	10.0-89.1	33.2	41.6 (36.0-47.3)	798.8 (p<0.001)	94.4 (93.2-95.3)	9.8-77.8
Women	22	2,078	14.8-89.1	49.7	47.7 (37.5-57.9)	426.5 (p<0.001)	95.1 (93.6-96.2)	6.2-91.2
Men	19	4,110	10.0-75.9	31.3	32.2 (26.5-38.2)	173.9 (p<0.001)	89.7 (85.3-92.7)	10.9-58.2
Mixed sexes	5	422	14.0-85.7	55.2	54.1 (27.8-79.3)	102.0 (p<0.001)	96.1 (93.2-97.7)	0.0-100
HIV-positive individuals and individuals in HIV-discordant couples	29	5,476	13.3-80.0	47.2	46.0 (39.2-52.9)	611.3 (p<0.001)	95.4 (94.3-96.3)	13.3-80.7
Women	7	889	46.4-80.0	63.6	66.2 (51.1-79.8)	82.0 (p<0.001)	92.7 (87.4-95.7)	16.9-99.7
Men	14	1,394	23.3-69.2	47.9	47.6 (37.1-58.2)	153.9 (p<0.001)	91.6 (87.6-94.3)	10.4-86.4
Mixed sexes	8	3,193	13.3-53.9	31.1	29.6 (22.6-37.0)	117.8 (p<0.001)	94.1 (90.5-96.3)	8.2-57.3
Other populations^e^	13	2,799	5.4-61.9	34.8	30.3 (21.2-40.2)	327.2 (p<0.001)	96.3 (95.0-97.3)	2.3-70.9
Women	6	776	15.2- 61.9	45.1	43.0 (30.0-56.5)	58.8 (p<0.001)	91.5 (84.3-95.4)	5.3-86.4
Men	6	2,008	5.4- 36.4	16.6	19.0 (9.7-30.5)	172.2 (p<0.001)	97.1 (95.5-98.1)	0.0-65.3
Mixed sexes	1^d^	15	-	-	40.0 (16.3-66.2)	-	-	-

Abbreviations: CI = Confidence interval, FSWs = Female sex workers, HIV = Human immunodeficiency virus, HSV-2 = Herpes simplex virus type 2, MSM = Men who have sex with men, MSWs = Male sex workers, STI = Sexually transmitted infection.

a Q: The Cochran's Q statistic is a measure assessing the existence of heterogeneity in pooled outcome measures, here HSV-2 seroprevalence.

b I^2^: A measure that assesses the magnitude of between-study variation that is due to actual differences in HSV-2 seroprevalence across studies rather than chance.

c Prediction interval: A measure that estimates the distribution (95% interval) of true HSV-2 seroprevalence around the estimated mean.

d No meta-analysis was done due to the small number of studies (n<3).

e Other populations include populations with an undetermined risk of acquiring HSV-2 infection such as patients with cervical cancer.

Pooled means of HSV-2 seroprevalence across populations and subpopulations are shown in Tables 1 and 2. Pooled mean HSV-2 seroprevalence was lowest at 12.1% (95% confidence interval (CI):11.0-13.2%) among general populations, followed by 20.2% (95% CI: 17.4-23.1%) among intermediate-risk populations, 41.6% (95% CI: 36.0-47.3%) among STI clinic attendees and symptomatic populations, 46.0% (95% CI: 39.2-52.9%) among HIV-positive individuals and individuals in HIV-discordant couples, and 47.9% (95% CI: 44.2-51.6%) among higher-risk populations. Pooled mean seroprevalence was 62.2% (95% CI: 58.9-65.6%) in FSWs, but only 23.6% (95% CI: 20.9-26.3%) among men who have sex with men (MSM), male sex workers (MSWs), and transgender people. Across all populations, women had higher pooled mean seroprevalence than men (Table 1).

**Table 2:** Pooled mean estimates for HSV-2 seroprevalence in the general populations in Asia.

Population classification	Outcome measures	Sample size	HSV-2 seroprevalence (%)		Pooled mean HSV-2 seroprevalence	Heterogeneity measures		
	Total n	Total N	Range	Median	Mean (%) (95% CI)	Q^a^ (p-value)	I²^b^ (%) (95% CI)	Prediction interval^c^ (%)
**Countries**								
China	61	41,061	1.1-54.9	8.5	9.3 (7.8-10.9)	1,455.4 (p<0.001)	95.9 (95.2-96.4)	1.1-23.7
India	90	40,432	0.0-30.0	9.3	9.7 (8.6-10.9)	1,034.8 (p<0.001)	91.4 (90.0-92.6)	2.3-21.0
Japan	36	2,313	0.0-43.8	6.1	5.9 (3.9-8.2)	120.6 (p<0.001)	71.0 (59.4-79.3)	0.0-20.0
Papua New Guinea	14	2,482	6.2-71.0	30.5	30.1 (22.8-37.8)	162.1 (p<0.001)	92.0 (88.3-94.5)	6.2-61.7
South Korea	24	4,765	1.7-45.5	19.4	20.3 (14.9-26.1)	507.2 (p<0.001)	95.5 (94.2-96.4)	1.0-53.7
Sri Lanka	14	3,052	3.6-26.6	11.8	13.5 (9.8-17.7)	134.7 (p<0.001)	90.3 (85.6-93.5)	1.9-33.0
Taiwan	24	747	0.0-42.1	13.8	12.0 (7.3-17.5)	90.7 (p<0.001)	74.6 (62.2-83.0)	0.0-41.4
Vietnam	13	3,514	6.5-51.5	11.1	19.4 (12.1-27.9)	392.9 (p<0.001)	96.9 (95.9-97.7)	0.0-58.3
Other countries^d^	19	5,822	3.6-83.3	11.8	20.9 (14.5-28.2)	556.2 (p<0.001)	96.8 (95.9-97.5)	0.4-57.6
**Subregions**								
East Asia	145	48,886	0.0-54.9	8.6	10.5 (9.0-12.1)	3,531.8 (p<0.001)	95.9 (95.5-96.3)	0.0-32.8
South Asia	106	45,827	0.0-30.0	9.8	10.2 (9.2-11.3)	1,236.2 (p<0.001)	91.5 (90.3-92.6)	2.5-21.9
Southeast Asia	30	6,993	5.8-83.3	12.3	21.2 (15.8-27.1)	878.8 (p<0.001)	96.7 (96.0-97.3)	0.5-58.1
Papua New Guinea	14	2,482	6.2-71.0	30.5	30.1 (22.8-37.8)	162.1 (p<0.001)	92.0 (88.3-94.5)	6.2-61.7
**Age group**								
<20 years	15	3,729	0.0-9.6	5.4	4.7 (3.3-6.3)	37.2 (p<0.001)	62.4 (34.3-78.5)	1.0-10.3
20-29 years	48	13,118	0.0-27.5	6.4	6.5 (4.8-8.3)	616.3 (p<0.001)	92.4 (90.7-93.7)	0.0-21.4
30-39 years	45	10,859	0.0-30.9	10.4	9.3 (7.4-11.5)	504.4 (p<0.001)	91.3 (89.2-93.0)	0.5-25.7
40-49 years	35	8,551	0.0-34.5	9.6	10.5 (7.6-13.8)	626.6 (p<0.001)	94.6 (93.3-95.6)	0.0-33.6
50-59 years	13	1,173	8.5-38.4	15.4	16.2 (10.7-22.5)	78.4 (p<0.001)	84.7 (75.4-90.5)	0.9-42.7
≥60 years	13	870	10.1-51.5	25.8	26.6 (19.2-34.7)	73.6 (p<0.001)	83.7 (73.5-90.0)	4.0-58.7
Mixed	126	65,888	0.0-83.3	12.8	15.7 (13.9-17.7)	4,800.4 (p<0.001)	97.4 (97.2-97.6)	1.4-40.0
**Year of data collection category**								
≤2000	94	14,929	0.0-51.5	10.5	12.3 (10.1-14.6)	1,358.2 (p<0.001)	93.2 (92.2-94.0)	0.0-37.9
2001-2010	152	51,051	0.0-58.5	11.0	12.0 (10.7-13.3)	2,532.3 (p<0.001)	94.0 (93.4-94.6)	1.5-29.6
>2010	49	38,208	0.0-83.3	6.9	11.9 (9.3-14.6)	2,475.7 (p<0.001)	98.1 (97.8-98.3)	0.3-34.8
**Year of publication category**								
≤2005	83	12,855	0.0-44.9	10.4	11.9 (9.7-14.3)	1,130.4 (p<0.001)	92.7 (91.6-93.7)	0.0-37.1
2006-2015	178	55,863	0.0-71.0	11.4	13.1 (11.7-14.4)	3,484.3 (p<0.001)	94.9 (94.4-95.4)	1.2-33.6
>2015	34	35,470	0.0-83.3	4.9	7.9 (5.9-10.3)	1,503.8 (p<0.001)	97.8 (97.4-98.1)	0.1-24.5
**All studies**	**295**	**104,188**	**0.0-83.3**	**10.2**	**12.1 (11.0-13.2)**	**7,265.2 (p<0.001)**	**96.0 (95.7-96.2)**	**0.5-33.6**

Abbreviations: CI = Confidence interval, HSV-2 = Herpes simplex virus type 2.

a Q: The Cochran's Q statistic is a measure assessing the existence of heterogeneity in pooled outcome measures, here HSV-2 seroprevalence.

b I^2^: A measure that assesses the magnitude of between-study variation that is due to actual differences in HSV-2 seroprevalence across studies rather than chance.

c Prediction interval: A measure that estimates the distribution (95% interval) of true HSV-2 seroprevalence around the estimated mean.

d Other countries includes countries with <10 seroprevalence measures: Bangladesh, Indonesia, Philippines, and Thailand.

Among general populations, pooled mean seroprevalence varied by subpopulation (Table 2). Pooled mean seroprevalence increased steadily with age from 4.7% (95% CI: 3.3-6.3%) in individuals <20 years old (n=15), followed by 6.5% (95% CI: 4.8-8.3%) in those 20-29 years old (n=48), 9.3% (95% CI: 7.4-11.5%) in those 30-39 years old (n=45), 10.5% (95% CI: 7.6-13.8%) in those 40-49 years old (n=35), 16.2% (95% CI: 10.7-22.5%) in those 50-59 years old (n=13), and 26.6% (95% CI: 19.2-34.7%) in those ≥60 years old (n=13).

All meta-analyses showed evidence of heterogeneity (p-value<0.001) with wide prediction intervals and I^2^>50% indicating that most variation is *true* variation in seroprevalence rather than sampling variation (chance) (Tables 1 and 2). Forest plots of different meta-analyses are presented in Figure S1.

### Associations between HSV-2 seroprevalence and population and study characteristics

Results of univariable and multivariable meta-regression analyses are in Table 3. Multivariable analyses were conducted to investigative associations with higher seroprevalence and sources of heterogeneity. Due to collinearity between temporal variables, two multivariable models were conducted, one including year of data collection as a categorical variable and one including it as a linear term.

**Table 3:** Univariable and multivariable meta-regression analyses for HSV-2 seroprevalence in Asia.

			Outcome measures	Sample size	Univariable analysis				Multivariable analysis			
	Total n	Total N	*RR* (95%CI)	p-value	LR test p-value		Adjusted R^2^ (%)		Model 1^a^		Model 2^b^	
									*ARR* (95% CI)	p-value	*ARR* (95% CI)	p-value
**Population characteristics**	**Population type**	General populations	295	104,188	1.00	-	<0.001	40.30	1.00	-	1.00	-
		Intermediate-risk populations	86	23,946	1.58 (1.32-1.85)	<0.001			1.81 (1.51-2.18)	<0.001	1.84 (1.54-2.20)	<0.001
		Higher-risk populations	209	73,139	3.40 (2.99-3.87)	<0.001			3.54 (3.10-4.03)	<0.001	3.53 (3.10-4.03)	<0.001
		STI clinic attendees and symptomatic populations	46	6,610	3.19 (2.54-3.99)	<0.001			2.37 (1.95-2.89)	<0.001	2.33 (1.91-2.84)	<0.001
		HIV-positive individuals and individuals in HIV-discordant couples	29	5,476	3.63 (2.77-4.75)	<0.001			3.57 (2.82-4.52)	<0.001	3.57 (2.82-4.51)	<0.001
		Other populations^c^	13	2,799	2.34 (1.56-3.49)	<0.001			2.06 (1.48-2.85)	<0.001	2.11 (1.52-2.93)	<0.001
	**Age group**	<20 years	21	4,257	1.00	-	<0.001	16.01	1.00	-	1.00	-
		20-29 years	63	16,222	1.10 (0.69-1.73)	0.695			1.20 (0.87-1.67)	0.265	1.17 (0.84-1.62)	0.338
		30-39 years	57	12,163	1.32 (0.83-2.10)	0.236			1.64 (1.18-2.28)	0.003	1.58 (1.13-2.19)	0.006
		40-49 years	40	9,111	1.45 (0.89-2.36)	0.131			1.91 (1.35-2.70)	<0.001	1.80 (1.27-2.55)	0.001
		50-59 years	14	1,620	1.76 (0.96-3.24)	0.067			2.73 (1.76-4.25)	<0.001	2.69 (1.73-4.19)	<0.001
		≥60 years	14	1,326	2.77 (1.52-5.04)	0.001			3.99 (2.60-6.15)	<0.001	4.03 (2.62-6.21)	<0.001
		Mixed	469	171,459	2.93 (1.95-4.40)	<0.001			1.77 (1.32-2.38)	<0.001	1.67 (1.24-2.24)	0.001
	**Sex**	Women	342	115,383	1.00	-	<0.001	8.24	1.00	-	1.00	-
		Men	306	91,939	0.61 (0.53-0.70)	<0.001			0.59 (0.53-0.65)	<0.001	0.58 (0.53-0.64)	<0.001
		Mixed sexes	30	8,836	1.14 (0.82-1.58)	0.437			1.23 (0.98-1.54)	0.077	1.21 (0.96-1.52)	0.100
	**Subregions**	East Asia	268	100,501	1.00	-	<0.001	3.10	1.00	-	1.00	-
		South Asia	309	96,357	1.42 (1.22-1.65)	<0.001			2.38 (1.78-3.18)	<0.001	2.42 (1.81-3.23)	<0.001
		Southeast Asia	83	16,554	1.45 (1.16-1.82)	0.001			1.57 (1.28-1.92)	<0.001	1.54 (1.25-1.90)	<0.001
		Papua New Guinea	18	2,746	1.82 (1.18-2.81)	0.006			4.75 (3.21-7.03)	<0.001	4.55 (3.08-6.72)	<0.001
	**National income**	LMIC	369	106,572	1.00	-	0.004	1.32	1.00	-	1.00	
		UMIC	185	97,549	0.76 (0.65-0.90)	0.001			1.66 (1.28-2.17)	<0.001	1.66 (1.28-2.17)	<0.001
		HIC	124	12,037	0.87 (0.71-1.06)	0.167			2.49 (1.86-3.34)	<0.001	2.35 (1.75-3.16)	<0.001
**Study methodology characteristics**	**Assay type**	Western Blot	44	4,884	1.00	-	0.200	0.23	-	-	-	-
		ELISA	634	211,274	0.82 (0.61-1.11)	0.200			-	-	-	-
	**Sample size**^d^	<200	185	9,414	1.00	-	<0.001	14.57	1.00	-	1.00	-
		≥200	493	206,744	0.48 (0.41-0.56)	<0.001			0.76 (0.67-0.87)	<0.001	0.77 (0.68-0.89)	<0.001
	**Sampling method**	Probability based	298	100,245	1.00	-	0.032	0.60	1.00	-	1.00	-
		Non-probability based	380	115,913	1.17 (1.01-1.35)	0.032			1.20 (1.06-1.35)	0.003	1.20 (1.06-1.35)	0.003
	**Response rate**	≥80%	129	53,942	1.00	-	<0.001	8.83	1.00	-	1.00	-
		<80%	165	28,416	2.19 (1.79-2.69)	<0.001			0.92 (0.78-1.08)	0.311	0.98 (0.83-1.16)	0.871
		Unclear	384	133,800	1.50 (1.25-1.79)	<0.001			0.88 (0.77-1.01)	0.076	0.90 (0.78-1.03)	0.148
**Temporal variables**	**Year of data collection category**	≤2000	153	25,288	1.00	-	0.003	1.57	1.00	-	-	-
		2001-2010	415	121,894	1.17 (0.98-1.39)	0.089			0.82 (0.72-0.93)	0.003	-	-
		>2010	110	68,976	0.84 (0.67-1.06)	0.141			0.65 (0.55-0.77)	<0.001	-	-
	**Year of data collection**	678	216,158	0.99 (0.98-1.01)	0.345	0.345	0.18	-	-	0.98 (0.97-0.99)	<0.001	

Abbreviations: ARR = Adjusted risk ratio, CI = Confidence interval*,* ELISA = Enzyme-linked immunosorbent type-specific assay, HIC = High-income country, HIV = Human immunodeficiency virus, HSV-2 = Herpes simplex virus type 2, LMIC = Lower-middle-income country, LR = Likelihood ratio, RR = Risk ratio, STI = Sexually transmitted infection, UMIC = Upper-middle-income country.

a Variance explained by multivariable model 1 (adjusted *R^2^*) = 65.16%.

b Variance explained by multivariable model 2 (adjusted *R^2^*) = 64.96%.

c Other populations include populations with an undetermined risk of acquiring HSV-2 infection such as patients with cervical cancer.

d Sample size denotes the sample size of each study population at the baseline found in the original publication, based on the original reported study design (case control, cross sectional, cohort, or randomized controlled trial).

The first model, including “year of data collection” as a categorical variable, explained 65.16% of seroprevalence variation (Table 3). Compared to general populations, HSV-2 seroprevalence was highest in HIV-positive individuals and individuals in HIV-discordant couples with an adjusted risk ratio (ARR) of 3.57 (95% CI: 2.82-4.52), followed by higher-risk populations, STI clinic attendees and symptomatic populations, and intermediate-risk populations.

Compared to individuals <20 years of age, seroprevalence increased steadily with age and was highest among individuals ≥60 years of age. Men had a lower seroprevalence than women with an ARR of 0.59 (95% CI: 0.53-0.65), indicating that seroprevalence among women was nearly 70% (inverse of ARR) higher than that among men. Seroprevalence was lowest in East Asia, followed by Southeast Asia, South Asia, and was highest in Papua New Guinea. Seroprevalence increased with national income.

Studies with larger sample sizes reported lower seroprevalence and studies using non-probability sampling reported higher seroprevalence. Seroprevalence did not vary by the assay type or reported response rate.

Data collected between 2001-2010 and after 2010 had lower seroprevalence than those collected before 2000 with an ARR of 0.82 (95% CI: 0.72-0.93) and 0.65 (95% CI: 0.55-0.77), respectively.

The second model, including year of data collection as a linear term, explained 64.96% of seroprevalence variation and yielded results similar to those of the first model. Seroprevalence declined over time with an ARR of 0.98 (95% CI: 0.97-0.99) per year (Table 3), that is a 2% decline per year in the last three decades.

Sensitivity analyses were conducted including “year of publication” in lieu of “year of data collection”, both as a categorical variable and as a linear term. The two analyses yielded similar results (Table S8).

### HSV-2 isolation in GUD and in genital herpes

Identified proportions of HSV-2 isolation in GUD and in genital herpes are listed in Table S9 and summarized in Table 4. Large proportion of studies reporting GUD (n=14) and genital herpes (n=27) outcome measures were published before 2005 (n=6, 42.9%; and n=20, 74.1%; respectively) (Table S9). In GUD cases (n=15), the proportion of HSV-2 isolation ranged from 4.6-100.0%, with a median of 44.4% and a pooled mean proportion of 48.2% (95% CI: 34.9-61.6%) (Table 4). Among women (n=3), the proportion ranged from 36.4-100%, with a median of 71.8% and a pooled mean of 73.2% (95% CI: 36.2-98.0%). Among men (n=5), it ranged from 4.6-100%, with a median of 26.7% and a pooled mean of 34.1% (95% CI: 15.8-55.1%).

**Table 4:** Pooled mean proportions of HSV-2 virus in clinically diagnosed cases of genital ulcer disease and genital herpes in Asia.

Population type	Outcome measures	Samples	Proportion of HSV-2 isolation (%)	Pooled proportion of HSV-2 isolation (%)	Heterogeneity measures			
	Total n	Total N	Range	Median	Mean (95% CI)	Q^a^ (p-value)	I²^b^ (%) (95% CI)	Prediction Interval^c^ (%)
Patients with GUD	15	1,673	4.6-100	44.4	48.2 (34.9-61.6)	373.7 (p<0.001)	96.3 (95.0-97.2)	3.2-95.2
Women	3	159	36.4-100	71.8	73.2 (36.2-98.0)	38.74 (p<0.001)	94.8 (88.3-97.7)	0.0-100.0
Men	5	772	4.6-100	26.7	34.1 (15.8-55.1)	90.8 (p<0.001)	95.6 (92.2-97.5)	0.0-98.6
Mixed sexes	7	742	19.2-77.4	48.5	47.6 (25.8-69.9)	204.9 (p<0.001)	97.1 (95.6-98.1)	0.0-100.0
Patients with genital herpes	36	2,060	27.8-100	73.1	75.9 (68.3-82.8)	465.4 (p<0.001)	92.5 (90.6-94.0)	28.1-100.0
Women	14	458	27.8-100	69.1	69.2 (54.7-82.1)	122.7 (p<0.001)	89.4 (84.0-93.0)	13.3-100.0
Men	5	300	33.3-100	89.8	84.4 (63.3-97.9)	59.3 (p<0.001)	93.3 (87.2-96.4)	5.2-100.0
Mixed sexes	17	1,302	47.3-100	75.0	78.0 (68.4-86.3)	217.1 (p<0.001)	92.6 (89.7-94.7)	33.4-100.0

Abbreviations: CI = Confidence interval, GUD = Genital ulcer disease, HSV-2 = Herpes simplex virus type 2.

a Q: The Cochran's Q statistic is a measure assessing the existence of heterogeneity in pooled outcome measures, here proportions of HSV-2 virus isolation in GUD and in genital herpes.

b I^2^: A measure assessing the magnitude of between-study variation that is due to true differences in proportions of HSV-2 virus isolation across studies rather than sampling variation.

c Prediction interval: A measure quantifying the distribution (95% interval) of true proportions of HSV-2 virus isolation around the estimated pooled mean.

In genital herpes cases (n=36), the proportion of HSV-2 isolation ranged from 27.8-100.0%, with a median of 73.1% and a pooled mean proportion of 75.9% (95% CI: 68.3-82.8%) (Table 4). Among women (n=14), the proportion ranged from 27.8-100%, with a median of 69.1% and a pooled mean of 69.2% (95% CI: 54.7-82.1%). Among men (n=5), it ranged from 33.3-100%, with a median of 89.8% and a pooled mean of 84.4% (95% CI: 63.3-97.9%).

Heterogeneity was evident in all meta-analyses (p-value<0.001, I^2^>50%, and wide prediction intervals). Forest plots are shown in Figure S2.

### Quality assessment

The quality assessment of the 272 seroprevalence studies are summarized in Table S10. Briefly, 209 studies (76.8%) had high precision, 74 studies (27.2%) had low ROB in the sampling method domain, and 50 studies (18.4%) had low ROB in the response rate domain. Only 23 studies (8.4%) had low ROB in both quality domains, and only 24 studies (8.8%) had high ROB in both quality domains.

## Discussion

This study provided a thorough, systematic investigation of HSV-2 epidemiology in Asia. HSV-2 seroprevalence was estimated at 12% of the general population of this region, lower than that estimated recently in Africa at 37%^24^ and in Latin America and the Caribbean (LAC) at 21%.^39^ Remarkably, seroprevalence has been declining at about 2% per year over the last three decades, just as recently found in Africa,^24^ LAC,^39^ and the United States of America.[40], [41], [42], [43] Such similar declines in different regions may suggest that risky sex has declined since recognition of the HIV epidemic,[44], [45], [46], [47] increased STI awareness,^48^ greater access to HIV/STI services,^22^^,^^49^ and/or possibly socio-economic changes that affected the structure of sexual networks leading to lower infection incidence. The declining HSV-2 seroprevalence is also consistent with observed declines in different regions, including Asia, in the prevalence of HIV[50], [51], [52] and syphilis,^53^ which could not be explained by higher treatment coverage. It remains to be seen whether these declines are specific to certain regions or subregions, or whether they are global, since detailed analyses for all regions are not yet available.

HSV-2 persists as the etiological cause of nearly half (48%) of GUD cases, similar to Africa at 51%^24^ and LAC at 41%.^39^ The disproportional role of HSV-2 infection in this disease outcome may persist for decades despite its declining seroprevalence, as other causes, such as syphilis,^53^ may also be declining concurrently. Interestingly, the role of HSV-2 in GUD appeared higher among women than men (Table 4), probably because of the higher HSV-2 seroprevalence in women and possibly a higher incidence of other ulcerative STIs among men, perhaps as a consequence of more risky sexual behaviour among men.

HSV-2 infection (as opposed to HSV-1 infection) proved to be the etiological cause of 76% of genital herpes cases, lower than in Africa at 97%^24^ and in LAC at 91%.^39^ This finding is consistent with the finding that HSV-1 infection now plays a growing role in genital herpes in Asia,^25^ just as in North America^40^^,^^54^ and Europe,[55], [56], [57], [58], [59] but differs from the pattern found in Africa,^60^ LAC,^61^ and possibly the Middle East and North Africa,^62^ where sexual transmission of HSV-1 infection is still less pervasive. Curiously, HSV-2 seems more significant in genital herpes among men than women (Table 4), a finding that may reflect the relative ease of clinically diagnosing this condition in men compared to women.

Results confirmed key attributes of HSV-2 epidemiology that appear across Asia, Africa,^24^ and LAC.^39^ Seroprevalence varied immensely by population sexual-risk classification,^24^^,^^39^ and this heterogeneity explained nearly half the seroprevalence variation across studies (Table 3). Seroprevalence was very high in populations such as FSWs, HIV patients, and STI clinic attendees (Table 1). FSWs had a much higher seroprevalence at 62% than MSM at 23% (Table 1), and women had nearly 70% higher seroprevalence than men (Table 3),^24^^,^^39^ all affirming women's higher bio-anatomical susceptibility to this infection,^63^^,^^64^ and consistent with what is observed in other regions.^24^^,^^39^ Seroprevalence increased steadily with age starting from sexual debut and consistent with increasing cumulative risk of exposure over sexual lifespan (Table 3).^24^^,^^39^ Subregional variability in seroprevalence exists within each global region.^24^^,^^39^ In Asia, Papua New Guinea had the highest seroprevalence at 30% (Table 2), consistent with higher levels of STIs.^65^ However, the role of national income (proxy for the average socio-economic condition in the country) appears to vary from one global region to another. Higher national income was associated with lower seroprevalence in LAC,^39^ but higher seroprevalence in Asia (Table 3).

The present study had some limitations. No data were available for 8 of the 25 countries/territories in this region (Macao, Mongolia, North Korea, Nepal, Brunei, Cambodia, Lao, and Myanmar). Studies varied in methods and quality, and used different diagnostic assays. Many did not use probability-based sampling. There was evidence that use of non-probability sampling and smaller sample size (<200) affected the measured seroprevalence, yet no effect was found for assay type or response rate (Table 3). A large proportion of studies reporting GUD and genital herpes measures were published before 2005; thus, they may not be representative of the current contribution of HSV-2 to GUD and genital herpes. Publication bias was not assessed because of methodological issues in assessing it for proportion measures.^66^ The study did not attempt to link specific prevention strategies to observed patterns. Despite these limitations and the existence of heterogeneity, a large volume of data was available to enable a range of analyses and 65% of the observed heterogeneity among studies was subsequently explained through meta-regressions (Table 3). Therefore, these limitations did not pose a barrier to interpretation of the study results or its findings.

## Conclusions

Approximately 1 in 10 individuals is chronically infected with HSV-2 in Asia, but seroprevalence is declining at 2% per year. Despite this decline, it will take at least a generation before there will be a major change in seroprevalence. HSV-2 infection appears to be the etiological cause of half of GUD cases and three-quarters of genital herpes cases. These findings support the need for a fundamental intervention such as a vaccine, as well as universal access to sexual and reproductive health services. There is a need to expand and broaden HSV-2 research and surveillance in this region by conducting repeated population-based seroprevalence surveys, actively monitoring clinical cases of GUD and genital herpes and their aetiologies, and accelerating ongoing efforts to develop prophylactic and therapeutic HSV-2 vaccines.^20^^,^^21^^,^^67^

## Declaration of Competing Interest

The authors declare no competing interests.

## Appendix Supplementary materials

Image, application 1

Image, application 2
