# Supplementary material for: Epidemiology of herpes simplex virus type 2 in Asia: A systematic review, meta-analysis, and meta-regression
Source: Lancet Reg Health West Pac. 2021 Jun 9;12:100176. doi: 10.1016/j.lanwpc.2021.100176 (PMC8356094; doi:10.1016/j.lanwpc.2021.100176)
Supplement: Supplementary file 1 [file mmc1.docx]

**Supplementary Material**

**Epidemiology of herpes simplex virus type 2 in Asia: A systematic review, meta-analysis, and meta-regression**

Sawsan AlMukdad MSc,^a,b^ Manale Harfouche MPH,^a,b^ Anja Wettstein MSc,^a^ and Laith J. Abu-Raddad PhD^a,b,c^

*^a^ Infectious Disease Epidemiology Group, Weill Cornell Medicine-Qatar, Cornell University, Qatar Foundation - Education City, Doha, Qatar*

*^b^ World Health Organization Collaborating Centre for Disease Epidemiology Analytics on HIV/AIDS, Sexually Transmitted Infections, and Viral Hepatitis, Weill Cornell Medicine–Qatar, Cornell University, Qatar Foundation – Education City, Doha, Qatar*

^c^ Department of Population Health Sciences, Weill Cornell Medicine, Cornell University, New York, New York, USA

Table of Contents

[**Supplementary Table S1.** Preferred Reporting Items for Systematic Reviews and Meta-analyses (PRISMA) checklist 1.^1^ 3](#_Toc69312051)

[**Supplementary Table S2.** Data sources and search criteria for systematically reviewing HSV-2 epidemiology in Asia. 5](#_Toc69312052)

[**Supplementary Box S1.** The 26 countries/territories included in our definition of Asia.^2^ 6](#_Toc69312053)

[**Supplementary Box S2.** Variables extracted from relevant reports meeting the inclusion criteria. 7](#_Toc69312054)

[**Supplementary Box S3.** Definitions of population type classifications. 8](#_Toc69312055)

[**Supplementary Box S4.** Factors (variables) selected *a priori* and included in univariable and multivariable meta-regression analyses. 9](#_Toc69312056)

[**Supplementary Table S3.** Studies reporting HSV-2 seroincidence in Asia 10](#_Toc69312057)

[**Supplementary Table S4.** Studies reporting HSV-2 seroprevalence in East Asia. 11](#_Toc69312058)

[**Supplementary Table S5.** Studies reporting HSV-2 seroprevalence in South Asia. 14](#_Toc69312059)

[**Supplementary Table S6.** Studies reporting HSV-2 seroprevalence in Southeast Asia among different populations. 17](#_Toc69312060)

[**Supplementary Table S7.** Studies reporting HSV-2 seroprevalence in Papua New Guinea. 19](#_Toc69312061)

[**Supplementary Figure S1.** Forest plots presenting outcomes of the pooled mean HSV-2 seroprevalence among different populations in Asia. 20](#_Toc69312062)

[A) General populations 20](#_Toc69312063)

[B) Intermediate-risk populations 24](#_Toc69312064)

[C) Higher-risk populations 25](#_Toc69312065)

[D) STI clinic attendees and symptomatic populations 28](#_Toc69312066)

[E) HIV-positive individuals and individuals in HIV-discordant couples 29](#_Toc69312067)

[F) Other populations 30](#_Toc69312068)

[**Supplementary Table S8.** Univariable and multivariable meta-regression analyses for HSV-2 seroprevalence in Asia using the year of publication instead of the year of data collection as the time variable. 31](#_Toc69312069)

[**Supplementary Table S9.** Studies reporting proportions of HSV-2 virus isolation in clinically diagnosed genital ulcer disease and in clinically diagnosed genital herpes in Asia. 32](#_Toc69312070)

[**Supplementary Figure S2.** Forest plots presenting outcomes of the pooled mean proportions of HSV-2 virus isolation in clinically diagnosed genital ulcer disease and in clinically diagnosed genital herpes in Asia. 33](#_Toc69312071)

[A) Patients with genital ulcer disease 33](#_Toc69312072)

[B) Patients with genital herpes 34](#_Toc69312073)

[**Supplementary Table S10.** Summary of precision assessment and risk of bias (ROB) assessment for studies reporting HSV-2 seroprevalence in Asia. 35](#_Toc69312074)

[**References** 36](#_Toc69312075)

# **Supplementary Table S1.** Preferred Reporting Items for Systematic Reviews and Meta-analyses (PRISMA) checklist 1.^1^

| **Section/topic** | **#** | **Checklist item** | **Reported in main text on** |
| --- | --- | --- | --- |
| **Title** | | |  |
| Title | 1 | Identify the report as a systematic review, meta-analysis, or both. | p. 1 |
| **Abstract** | | |  |
| Structured summary | 2 | Provide a structured summary including, as applicable: background; objectives; data sources; study eligibility criteria, participants, and interventions; study appraisal and synthesis methods; results; limitations; conclusions and implications of key findings; systematic review registration number. | p. 2-3 |
| **Introduction** | | |  |
| Rationale | 3 | Describe the rationale for the review in the context of what is already known. | p. 6-7 |
| Objectives | 4 | Provide an explicit statement of questions being addressed with reference to participants, interventions, comparisons, outcomes, and study design (PICOS). | p. 6-7 |
| **Methods** | | |  |
| Protocol and registration | 5 | Indicate if a review protocol exists, if and where it can be accessed (e.g., Web address), and, if available, provide registration information including registration number. | NA |
| Eligibility criteria | 6 | Specify study characteristics (e.g., PICOS, length of follow-up) and report characteristics (e.g., years considered, language, publication status) used as criteria for eligibility, giving rationale. | p. 7-8 |
| Information sources | 7 | Describe all information sources (e.g., databases with dates of coverage, contact with study authors to identify additional studies) in the search and date last searched. | p. 7 |
| Search | 8 | Present full electronic search strategy for at least one database, including any limits used, such that it could be repeated. | Supplementary Table 2 |
| Study selection | 9 | State the process for selecting studies (i.e., screening, eligibility, included in systematic review, and, if applicable, included in the meta-analysis). | p. 7-8 |
| Data collection process | 10 | Describe method of data extraction from reports (e.g., piloted forms, independently, in duplicate) and any processes for obtaining and confirming data from investigators. | p. 8; Supplementary Box 2 |
| Data items | 11 | List and define all variables for which data were sought (e.g., PICOS, funding sources) and any assumptions and simplifications made. | p. 8; Supplementary Box 2 |
| Risk of bias in individual studies | 12 | Describe methods used for assessing risk of bias of individual studies (including specification of whether this was done at the study or outcome level), and how this information is to be used in any data synthesis. | p. 8-9 |
| Summary measures | 13 | State the principal summary measures (e.g., risk ratio, difference in means). | p. 9 |
| Synthesis of results | 14 | Describe the methods of handling data and combining results of studies, if done, including measures of consistency (e.g., I^2^) for each meta-analysis. | p. 9 |
| Risk of bias across studies | 15 | Specify any assessment of risk of bias that may affect the cumulative evidence (e.g., publication bias, selective reporting within studies). | p. 8-9 |
| Additional analyses | 16 | Describe methods of additional analyses (e.g., sensitivity or subgroup analyses, meta-regression), if done, indicating which were pre-specified. | p. 9 |
| **Results** | | |  |
| Study selection | 17 | Give numbers of studies screened, assessed for eligibility, and included in the review, with reasons for exclusions at each stage, ideally with a flow diagram. | p. 10; Figure 1 |
| Study characteristics | 18 | For each study, present characteristics for which data were extracted (e.g., study size, PICOS, follow-up period) and provide the citations. | p. 10-12; Supplementary Tables 3-7,9 |
| Risk of bias within studies | 19 | Present data on risk of bias of each study and, if available, any outcome level assessment (see item 12). | p. 13; Supplementary Table 10 |
| Results of individual studies | 20 | For all outcomes considered (benefits or harms), present, for each study: (a) simple summary data for each intervention group (b) effect estimates and confidence intervals, ideally with a forest plot. | p. 10-13; Tables 1,2 and 4; Supplementary Figures 1 and 2 |
| Synthesis of results | 21 | Present results of each meta-analysis done, including confidence intervals and measures of consistency. | p. 10-13;  Tables 1, 2 and 4 |
| Risk of bias across studies | 22 | Present results of any assessment of risk of bias across studies (see Item 15). | p. 13; Supplementary Table 10 |
| Additional analysis | 23 | Give results of additional analyses, if done (e.g., sensitivity or subgroup analyses, meta-regression [see Item 16]). | p. 11-12; Table 3; Supplementary Table 8 |
| **Discussion** | | |  |
| Summary of evidence | 24 | Summarize the main findings including the strength of evidence for each main outcome; consider their relevance to key groups (e.g., healthcare providers, users, and policy makers). | p. 13-14 |
| Limitations | 25 | Discuss limitations at study and outcome level (e.g., risk of bias), and at review-level (e.g., incomplete retrieval of identified research, reporting bias). | p. 15 |
| Conclusions | 26 | Provide a general interpretation of the results in the context of other evidence, and implications for future research. | p. 15 |
| **funding** | | |  |
| Funding | 27 | Describe sources of funding for the systematic review and other support (e.g., supply of data); role of funders for the systematic review. | p. 16 |

Abbreviations: NA = Not applicable, p = page.

# **Supplementary Table S2.** Data sources and search criteria for systematically reviewing HSV-2 epidemiology in Asia.

| **PubMed (last searched June 22, 2020):** |
| --- |
| (Simplexvirus[MeSH] OR Herpes Simplex[MeSH] OR Herpes Genitalis[MeSH] OR Herpes Hominis[Text] OR HSV type-2[Text] OR HSV type 2[Text] OR HSV2[Text] OR HSV-2[Text] OR HSV [Text] OR Human herpes virus[Text] OR Herpes simplex virus type 2[Text] OR Herpes simplex virus type-2[Text] OR herpes simplex virus 2[Text] OR herpes simplex virus-2[Text] OR herpes simplex type 2[Text] OR herpes simplex type-2[Text] OR herpes simplex 2[Text] OR herpes simplex-2[Text] OR Herpesvirus type 2[Text] OR Herpesvirus type-2[Text] OR Herpesvirus 2[Text] OR Herpesvirus-2[Text] OR Herpes virus type 2[Text] OR Herpes virus type-[Text] OR Herpes virus [Text] OR Herpes virus-2[Text] OR genital herpes[Text] OR Herpes Genitalis[Text] OR Stomatitis Herpetic[Text] OR Herpes Labialis[Text]) **AND**  (“Asia, Central”[MeSH] OR “Asia, Southeastern”[Mesh] OR “Bangladesh”[MeSH] OR “Bhutan”[MeSH] OR “India”[MeSH] OR “Nepal”[MeSH] OR “Sri Lanka”[MeSH] OR “Far East”[MeSH] OR “Papua New Guinea”[MeSH] OR Brunei*[Text] OR Cambodia*[Text] OR Indonesia*[Text] OR Lao*[Text] OR Malaysia*[Text] OR Myanmar[Text] OR Burm*[Text] OR Philippin*[Text] OR Singapor*[Text] OR Thai*[Text] OR Timor-Leste[Text] OR Timor*[Text] OR Vietnam*[Text] OR Bangladesh*[Text] OR Bhutan*[Text] OR India*[Text] OR Nepal*[Text] OR Sri Lanka*[Text] OR China[Text] OR Chinese[Text] OR Hong Kong[Text] OR Maca*[Text] OR Tibet*[Text] OR Japan*[Text] OR Korea*[Text] OR Mongolia*[Text] OR Taiwan*[Text] OR Papua New Guinea*[Text]) |
| **Embase (last searched June 22, 2020):** |
| (exp Herpes simplex/ or exp Herpesviridae/) OR (Herpes simplex or Herpes simplex virus or HSV type-2 or HSV type 2 or HSV2 or HSV-2 or HSV 2 or human herpes virus or Herpes simplex virus type 2 or Herpes simplex virus type-2 or herpes simplex virus 2 or herpes simplex virus-2 or herpes simplex type 2 or herpes simplex type-2 or herpes simplex 2 or herpes simplex-2 or Herpesvirus type 2 or Herpesvirus type-2 or Herpesvirus 2 or Herpesvirus-2 or Herpes virus type 2 or Herpes virus type-2 or Herpes virus 2 or Herpes virus-2 or genital herpes or Herpes Genitalis or herpes labialis or herpetic stomatitis).mp.) **AND** exp Far East/ or exp Bangladesh/ or exp Bhutan/ or exp India/ or exp Nepal/ or exp Sikkim/ or exp Sri lanka/ or exp Southeast Asia/ or (South* Asia or Bangladesh* or Bhutan* or India* or Nepal* or Sikkim* or Sri lanka* or Brunei* or Cambodia* or Indonesia* or Lao* or Malaysia* or Myanmar or Burm* or Papua new guinea* or Singapor* or Thai* or Timor* or Viet nam* or Vietnam* or China or Chinese or Philippin* or Hong Kong or Maca* or Tibet* or Japan* or Korea* or Mongolia* or Taiwan*).mp. |

Abbreviations: HSV-2 = Herpes simplex virus type 2

# **Supplementary Box S1.** The 26 countries/territories included in our definition of Asia.^2^

- **East Asia:** China, Hong Kong, Japan, Macao, Mongolia, North Korea, Taiwan, South Korea.
- **South Asia:** Bangladesh, Bhutan, India, Nepal, Sri Lanka.
- **Southeast Asia:** Brunei, Cambodia, Indonesia, Lao, Malaysia, Myanmar, Philippines, Singapore, Thailand, Timor-Leste, Vietnam.
- **Papua New Guinea**

# **Supplementary Box S2.** Variables extracted from relevant reports meeting the inclusion criteria.

1. Author(s)
2. Publication title
3. Publication year
4. Year(s) of data collection
5. Country of origin
6. Country of survey
7. City
8. Study site
9. Study design
10. Study sampling procedure
11. Study population
12. Population characteristics (e.g., sex and age)
13. Diagnostic assay
14. Sample size
15. HSV-2 outcome measures

Abbreviations: HSV-2 = Herpes simplex virus type 2

# **Supplementary Box S3.** Definitions of population type classifications.

| 1. **General populations** (populations at low risk): these include populations at lower risk of exposure to HSV-2, such as antenatal clinic attendees, blood donors, and pregnant women, among others. 2. **Intermediate-risk populations**: these include populations who presumably have frequent sexual contacts with populations engaging in high sexual risk behavior, and have therefore a higher risk of exposure to HSV-2 than the general population. These comprise prisoners, people who inject drugs, and truck drivers, among others. 3. **Higher-risk populations**: these include populations at high risk of exposure to HSV-2 as a consequence of specific sexual risk behaviors such as female sex workers, men who have sex with men, male sex workers, and transgender populations, among others. 4. **STI clinic attendees and symptomatic populations**: these include patients attending STI clinics, or have clinical manifestations related to an STI. 5. **HIV-positive individuals and individuals in HIV-discordant couples**: these include populations who are HIV-positive or are in a spousal relationship with an HIV-positive individual. 6. **Other populations**: these include populations not satisfying above definitions, or populations with an undetermined risk of acquiring HSV-2 infection. |
| --- |

Abbreviations: HSV-2 = Herpes simplex virus type 2, STI = Sexually transmitted infection, HIV = Human immunodeficiency virus.

# **Supplementary Box S4.** Factors (variables) selected *a priori* and included in univariable and multivariable meta-regression analyses.

| 1. Population type as defined in Box S3 2. Sex 3. Age groups classified to best fit reported data as:  - <20 years old - 20-29 years old - 30-39 years old - 40-49 years old - 50-59 years-old - ≥60 years old - Mixed age bands  1. Asia subregion as defined in Box S1 2. National income as classified by the World Bank^3^ 3. Assay type:  - Western Blot - ELISA  1. Sample size:  - <200 - ≥200  1. Sampling method:  - Probability-based sampling - Non-probability-based sampling  1. Response rate:  - ≥80% - <80% - Unclear  1. Year of publication category:  - ≤2005 - 2006-2015 - >2015  1. Year of publication as a linear term 2. Year of data collection category^*^  - ≤2000 - 2001-2010 - >2010  1. Year of data collection as a linear term |
| --- |

^*^ The categories were set based on the observed median time between the year of publication and year of data collection of 4 approximated to 5 to have 5-year brackets.

Abbreviations: ELISA = Enzyme-linked immunosorbent assay

**Supplementary Table S3.** Studies reporting HSV-2 seroincidence in Asia.

| **Author, year** | **Year(s) of data collection** | **Country** | **Study design** | **Population characteristics** | **HSV-2 serological assay** | **Sample size** | **Follow-up duration** | **Person-years of follow-up** | **HSV-2 seroconversion rate (%)** | **HSV-2 incidence rate (per 100 person-years)** |
| --- | --- | --- | --- | --- | --- | --- | --- | --- | --- | --- |
| **General populations** | | |  |  |  |  |  |  |  |  |
| Bogaerts, 2001^4^ | 1999-99 | Bangladesh | Cohort | Married women | ELISA | 186 | 1 year | 349.0 | 1.6 | 0.9 |
| Hochberg, 2015^5^ | 2004-11 | India | Cohort | Healthy adults | ELISA | 7,892 | 5.6 years | - | - | 2.6 |
| Madhivanan, 2011^6^ | 2005-06 | India | Cohort | 15-30 years old women | ELISA | 696 | 6 months | - | 5.2 | 10.4 |
| **Intermediate-risk populations** | | | |  |  |  |  |  |  |  |
| Sutcliffe, 2009^7^ | 2005-06 | Thailand | Cohort | Adult methamphetamine users | ELISA | 380 | 1 year | - | 4.0 | - |
| **Higher-risk populations** | | | | | | | | | | |
| Li, 2012^8^ | 2009-09 | China | Cohort | MSM | ELISA | 760 | 1 year | 570.6 | 6.1 | 8.1 |
| Thienkrua, 2016^9^ | 2006-10 | Thailand | Cohort | MSM | ELISA | 960 | 5 years | 2,866.0 | 13.3 | 4.5 |
| Wang, 2013^10^ | 2006-09 | China | Cohort | FSWs | ELISA | 334 | 3.5 years | - | 24.8 | 21.9 |
| **HIV-positive individuals and individuals in HIV-discordant couples** | | | | | | | | | | |
| Bollen, 2008^11^ | 1996-97 | Thailand | RCT | HIV-positive pregnant women | ELISA | 79 | 6 months | - | 8.9 | - |
| Ding, 2016^12^ | 2009-11 | China | Cohort | HIV-positive patients and their partners | ELISA | 930 | 1 year | 1301.8 | 5.2 | 3.8 |
| **STI clinic attendees and symptomatic populations** | | | | | | | | | | |
| Kumarasamy, 2008^13^ | 2002-03 | India | Cohort | >18 years old men | ELISA | 103 | 1 year | - | 12.0 | - |
| Kumarasamy, 2008^13^ | 2002-03 | India | Cohort | >18 years old women | ELISA | 107 | 1 year | - | 8.0 | - |
| Reynolds, 2003^14^ | 1993-00 | India | Cohort | Women attending an STI clinic | ELISA | 154 | - | 119.1 | 14.9 | 19.3 |
| Reynolds, 2004^15^ | 1993-00 | India | Cohort | Circumcised males | ELISA | 125 | - | 144.1 | 11.2 | 9.7 |
| Reynolds, 2004^15^ | 1993-00 | India | Cohort | Uncircumcised males | ELISA | 1,274 | - | 1,628.6 | 13.9 | 10.9 |
| Vallely, 2014^16^ | 2011-12 | Papua New Guinea | Cohort | Patients attending a sexual health clinic | ELISA | 83 | 1 year | - | 6.0 | 12.2 |

Abbreviations: ELISA = Enzyme-linked immunosorbent type-specific assay, FSWs = Female sex workers, HIV = Human immunodeficiency virus, HSV-2 = Herpes simplex virus type 2, MSM = Men who have sex with men, RCT = Randomized controlled trial, STI = Sexually transmitted infection.

# **Supplementary Table S4.** Studies reporting HSV-2 seroprevalence in East Asia.

| **Author, year** | **Year(s) of data collection** | **Country** | **Study site** | **Original study design^a^** | **Sampling method** | **Population** | **HSV-2 serological assay** | **Sample size** | **HSV-2 seroprevalence (%)** |
| --- | --- | --- | --- | --- | --- | --- | --- | --- | --- |
| **General populations** | | | | | | | | | |
| Ashley-Morrow, 2004^17^ | 2000-01 | South Korea | Community | CS | Conv | Adult women | WB | 97 | 43.3 |
| Chen, 2007^18^ | 2002 | China | Outpatient clinic | CS | Conv | Pregnant women | ELISA | 504 | 10.8 |
| Chen, 2019^19^ | 2016-17 | China | Outpatient clinic | Cohort | Conv | Women before pregnancy | ELISA | 10,669 | 4.7 |
| Doi, 2009^20^ | 2002 | Japan | Community | CS | RS | Healthy adults | ELISA | 1,244 | 8.4 |
| Guan, 2010^21^ | 2005-07 | China | Hospital | CC | Conv | Patients with acute myocardial infarction | ELISA | 102 | 54.9 |
| Guan, 2010^21^ | 2005-07 | China | Outpatient clinic | CC | RS | Healthy controls | ELISA | 150 | 38.0 |
| Hashido, 1998a^22^ | 1985-89 | Japan | Community | CS | Conv | Healthy adult women in Osaka | WB | 56 | 15.0 |
| Hashido, 1998a^22^ | 1985-89 | Japan | Community | CS | Conv | Female blood donors in Nagoya | WB | 19 | 0.0 |
| Hashido, 1998a^22^ | 1985-89 | Japan | Community | CS | Conv | Male blood donors in Nagoya | WB | 41 | 2.0 |
| Hashido, 1998a^22^ | 1985-89 | Japan | Community | CS | Conv | Pregnant women in Tokyo | WB | 90 | 7.0 |
| Hashido, 1998a^22^ | 1985-89 | Japan | Community | CS | Conv | Pregnant women in Kagoshima | WB | 200 | 17.0 |
| Hashido, 1999^23^ | 1973 | Japan | Community | CS | Conv | Adults sera collected in 1973,1983, and 1993 | ELISA | 614 | 5.0 |
| He, 2009^24^ | - | China | Community | CS | Conv | Male migrant workers | ELISA | 894 | 5.5 |
| Huai, 2019^25^ | 2016 | China | Community | CS | MSCS | General population in Shandong | ELISA | 7,256 | 3.5 |
| Itoh, 2000^26^ | 1993-99 | Japan | Hospital | CS | Conv | Women with retinal necrosis | EIA | 16 | 43.8 |
| Kim, 2003^27^ | 2000 | South Korea | Outpatient clinic | CS | Conv | Blood donors | ELISA | 200 | 5.0 |
| Lee, 2015^28^ | - | South Korea | Outpatient clinic | CS | Conv | General population | ELISA | 2,317 | 28.4 |
| Li, 2011^29^ | 2004-06 | China | Outpatient clinic | CS | Conv | Pregnant women | ELISA | 1,740 | 23.6 |
| Li, 2016a^30^ | 2013-14 | China | Community | CS | Conv | Healthy women before and during pregnancy | ELISA | 1,050 | 16.1 |
| Lin, 2011^31^ | 2006 | China | Community | CS | CRS | All residents of included rural villages | ELISA | 2,141 | 13.2 |
| Lo, 1999^32^ | 1995 | China | Outpatient clinic | CS | Conv | >25 years old females from general population | WB | 76 | 18.4 |
| Lo, 1999^32^ | 1995 | China | Outpatient clinic | CS | Conv | >25 years old males from general population | WB | 75 | 17.3 |
| Lo, 1999^32^ | 1995 | Hong Kong | Outpatient clinic | CS | Conv | Antenatal women in Hong Kong | WB | 78 | 12.8 |
| Lo, 1999^32^ | 1995 | China | Outpatient clinic | CS | Conv | Antenatal women in Southern China | WB | 62 | 3.2 |
| Nakagawa, 1991^33^ | - | Japan | Hospital | CS | Conv | Patients without malignancies | Immunodot | 14 | 36.0 |
| Peng, 1991^34^ | 1987-88 | China | Outpatient clinic | CC | Conv | Women without invasive cervical cancer | WB | 142 | 29.0 |
| Shen, 2015^35^ | 2007 | Taiwan | Community | CS | RS | General population from all age groups | ELISA | 1,072 | 7.7 |
| Shin, 2003^36^ | 1999-00 | South Korea | Community | CS | MSCS | Sexually active women | ELISA | 812 | 41.7 |
| Shin, 2007^37^ | 2004 | South Korea | Outpatient clinic | CS | Conv | <20 years old visitors of public health centers | ELISA | 1,194 | 2.8 |
| Shin, 2007^37^ | 2004 | South Korea | Outpatient clinic | CS | Conv | >20 years old visitors of public health centers | ELISA | 1,456 | 24.9 |
| Shin, 2007^37^ | 2005 | South Korea | Outpatient clinic | CS | SRS | General population | ELISA | 455 | 8.8 |
| Wang, 2019^38^ | 2015-17 | China | Outpatient clinic | Cohort | Conv | Women of reproductive age | ELISA | 13,672 | 6.1 |
| Yongjun, 2009^39^ | 2003-04 | China | Community | CS | MSCS | Married women | ELISA | 2,000 | 2.0 |
| Zhang, 2012^40^ | 2011 | China | Outpatient clinic | CS | Conv | Pregnant women with preeclampsia | ELISA | 52 | 3.9 |
| Zhang, 2012^40^ | 2011 | China | Outpatient clinic | CS | Conv | Healthy pregnant women | ELISA | 35 | 5.9 |
| Zhang, 2014^41^ | 2011 | China | Community | CS | RS | Healthy women | ELISA | 600 | 15.3 |
| **Intermediate-risk populations** | | | | | | | | | |
| Chen, 2006^42^ | 2000 | China | Community | CS | Conv | Truck drivers | ELISA | 550 | 4.4 |
| Reilly, 2012^43^ | 2010 | China | Community | CS | CRS | Male clients of FSWs | ELISA | 306 | 26.5 |
| Wei, 2004^44^ | 1999 | China | Community | CS | Conv | Hospitality girls | ELISA | 84 | 11.9 |
| Wu, 2007^45^ | - | China | Community | CS | Conv | All market vendors | ELISA | 4,510 | 6.5 |
| Xu, 2008^46^ | 2006 | China | Community | CS | Conv | Miners | ELISA | 1,424 | 8.4 |
| Xu, 2008^46^ | 2006 | China | Community | CS | Conv | Miners clients of FSWs | ELISA | 336 | 14.9 |
| Zhu, 2019^47^ | 2014-15 | China | Community | CS | TL & SS | Clients of FSWs | ELISA | 776 | 16.5 |
| **Higher-risk populations** | | | | | | | | | |
| Chen, 2005^48^ | 1999-00 | China | Outpatient clinic | CS | Conv | FSWs | ELISA | 505 | 65.1 |
| Chen, 2013^49^ | 2009 | China | Community | CS | Conv | FSWs working in sex venues | ELISA | 2,453 | 54.9 |
| Chen, 2015^50^ | 2009 | China | Community | CS | TL & SS | MSM who use drugs | ELISA | 177 | 16.9 |
| Chen, 2015^50^ | 2009 | China | Community | CS | TL & SS | MSM | ELISA | 649 | 12.8 |
| Ding, 2017^51^ | 2014 | China | VCT | CS | Conv | MSM | ELISA | 243 | 9.5 |
| Feng, 2010^52^ | 2007 | China | Community | CS | SS | MSM | ELISA | 538 | 24.7 |
| Gao, 2012^53^ | 2009 | China | Community | Cohort | Conv | MSM in Beijing | ELISA | 962 | 5.3 |
| Han, 2015^54^ | 2012 | China | Community | CS | Conv | FSWs | ELISA | 1,487 | 27.8 |
| Hashido, 1998a^22^ | 1985-89 | Japan | Community | CS | Conv | MSM in Nagoya | WB | 34 | 24.0 |
| Hashido, 1998a^22^ | 1985-89 | Japan | Community | CS | Conv | FSWs in Osaka | WB | 70 | 80.0 |
| Hu, 2017^55^ | 2009-14 | China | Outpatient clinic | CS | Conv | MSM | ELISA | 545 | 48.6 |
| Jiang, 2006^56^ | 2003 | China | Community | CS | Conv | MSM | ELISA | 90 | 7.8 |
| Kim, 2003^27^ | 2000 | South Korea | Outpatient clinic | CS | Conv | FSWs | ELISA | 200 | 71.0 |
| Li, 2016b^57^ | 2009-10 | China | Community | Cohort | Conv | MSM | ELISA | 962 | 5.3 |
| Liu, 2012a^58^ | 2009 | China | Community | CS | CRS | Money boys | ELISA | 418 | 11.0 |
| Liu, 2017a^59^ | 2013-14 | China | Community | CS | Conv | >18 years old MSM | ELISA | 486 | 22.4 |
| Liu, 2017b^60^ | 2015 | China | VCT | CS | Conv | MSM in Shanghai | ELISA | 732 | 5.6 |
| Lo, 1999^32^ | 1995 | China | Outpatient clinic | CS | Conv | FSWs | WB | 40 | 77.5 |
| Luo, 2015^61^ | 2009-12 | China | Community | CS | Conv | FSWs who were lost to follow up | ELISA | 1,096 | 60.4 |
| Luo, 2015^61^ | 2009-12 | China | Community | CS | Conv | FSWs who had at least one follow up | ELISA | 792 | 66.5 |
| Mao, 2018^62^ | 2012-13 | China | Community | CS | Conv | Younger MSM | ELISA | 1,289 | 7.6 |
| Mao, 2018^62^ | 2012-13 | China | Community | CS | Conv | Older MSM | ELISA | 3,131 | 14.5 |
| Ngo, 2008a^63^ | 2004 | China | Community | CS | Conv | CSWs | WB | 500 | 33.0 |
| Shin, 2007^37^ | 2003 | South Korea | Outpatient clinic | CS | Conv | Adult CSWs | ELISA | 304 | 81.6 |
| Wang, 2012a^64^ | 2006-09 | China | Community | CS | Conv | FSWs sera collected in 2006 (first survey) | ELISA | 741 | 67.3 |
| Wang, 2012a^64^ | 2006-09 | China | Community | CS | Conv | FSWs sera collected in 2006 (second survey) | ELISA | 407 | 67.8 |
| Wang, 2012a^64^ | 2006-09 | China | Community | CS | Conv | FSWs sera collected in 2007 (third survey) | ELISA | 705 | 70.8 |
| Wang, 2012a^64^ | 2006-09 | China | Community | CS | Conv | FSWs sera collected in 2007 (fourth survey) | ELISA | 255 | 54.5 |
| Wang, 2012a^64^ | 2006-09 | China | Community | CS | Conv | FSWs sera collected in 2008 (fifth survey) | ELISA | 587 | 68.3 |
| Wang, 2012a^64^ | 2006-09 | China | Community | CS | Conv | FSWs sera collected in 2008 (sixth survey) | ELISA | 281 | 63.4 |
| Wang, 2012a^64^ | 2006-09 | China | Community | CS | Conv | FSWs sera collected in 2009 (seventh survey) | ELISA | 548 | 70.6 |
| Wang, 2012a^64^ | 2006-09 | China | Community | CS | Conv | FSWs sera collected in 2009 (eighth survey) | ELISA | 251 | 60.2 |
| Wang, 2012b^65^ | 2009 | China | Community | CS | Conv | FSWs | ELISA | 345 | 58.3 |
| Wang, 2013^10^ | 2006-09 | China | Community | CS | Conv | FSWs | ELISA | 2,282 | 63.0 |
| Wei, 2004^44^ | 1999 | China | Community | CS | Conv | FSWs | ELISA | 101 | 29.7 |
| Xu, 2008^46^ | 2006 | China | Community | CS | Conv | FSWs | ELISA | 96 | 70.8 |
| Xu, 2011^66^ | 2006-07 | China | Community | Cohort | Conv | FSWs | ELISA | 1,642 | 66.9 |
| Xu, 2016^67^ | 2012 | China | Community | CS | TL & SS | MSM | ELISA | 4,415 | 12.5 |
| Yan, 2016^68^ | 2008 | China | Community | CS | RDS | MSM surveyed in 2008 | ELISA | 430 | 18.6 |
| Yan, 2016^68^ | 2012 | China | Community | CS | RDS | MSM surveyed in 2012 | ELISA | 589 | 10.2 |
| Yang, 2011^69^ | 2008-09 | China | Community | CS | Conv | FSWs | ELISA | 793 | 47.3 |
| Yao, 2012^70^ | 2007 | China | Community | CS | Conv | FSWs | ELISA | 397 | 67.4 |
| Yin, 2012^71^ | 2009-10 | China | Outpatient clinic | CS | Conv | MSM | ELISA | 962 | 5.3 |
| Yun, 2008^72^ | 2003 | South Korea | Outpatient clinic | CS | Conv | FSWs | ELISA | 1,487 | 27.8 |
| Zhang, 2013^73^ | 2008-09 | China | Community | CS | TL & SS | MSM | ELISA | 34 | 24.0 |
| Zhang, 2014^41^ | 2011 | China | Community | CS | RS | FSWs | ELISA | 70 | 80.0 |
| Zhu, 2008^74^ | - | China | Community | CS | Conv | MSM | ELISA | 545 | 48.6 |
| Zhu, 2017^75^ | 2014-15 | China | Community | CS | Conv | FSWs | ELISA | 90 | 7.8 |
| Zhu, 2018^76^ | 2014 | China | Community | CS | TL & SS | FSWs | ELISA | 200 | 71.0 |
| **HIV-positive individuals and individuals in HIV-discordant couples** | | | | | | | | | |
| Chen, 2010^77^ | 2004-07 | China | Outpatient clinic | CS | Conv | HIV-positive patients | ELISA | 195 | 13.3 |
| Ding, 2016^12^ | 2009-11 | China | Community | Cohort | Conv | HIV-positive partners | ELISA | 1,167 | 34.9 |
| Ding, 2016^12^ | 2009-11 | China | Community | Cohort | Conv | Individuals in an HIV-discordant couple | ELISA | 1,052 | 28.0 |
| Fu, 2009^78^ | - | China | Outpatient clinic | CS | Conv | HIV-positive patients | ELISA | 300 | 35.0 |
| He, 2011^79^ | 2008-09 | China | Outpatient clinic | CS | Conv | HIV-positive patients | ELISA | 1,110 | 34.1 |
| Kim, 2003^27^ | 2000 | South Korea | Outpatient clinic | CS | Conv | HIV-positive patients | ELISA | 200 | 65.0 |
| Shin, 2007^37^ | 2003 | South Korea | Outpatient clinic | CS | Conv | HIV-positive patients | ELISA | 196 | 48.5 |
| **STI clinic attendees and symptomatic populations** | | | | | | | | | |
| Hashido, 1997b^80^ | 1972-85 | Japan | Outpatient clinic | CS | Conv | Women with acute genital herpes | Immunodot | 30 | 36.7 |
| Hashido, 1997b^80^ | 1972-85 | Japan | Outpatient clinic | CS | Conv | Women with recurrent genital herpes | Immunodot | 25 | 88.0 |
| Hashido, 1997b^80^ | 1972-85 | Japan | Outpatient clinic | CS | Conv | Women with provoked genital herpes | Immunodot | 13 | 69.2 |
| Hashido, 1998a^22^ | 1985-89 | Japan | Outpatient clinic | CS | Conv | Male patients with STI in Osaka | WB | 26 | 23.0 |
| Kim, 2003^27^ | 2000 | South Korea | Outpatient clinic | CS | Conv | Overall STI clinic visitors | ELISA | 204 | 21.9 |
| Lo, 1999^32^ | 1995 | China | Outpatient clinic | CS | Conv | Females attending an STI clinic | WB | 76 | 35.5 |
| Lo, 1999^32^ | 1995 | China | Outpatient clinic | CS | Conv | Males attending an STI clinic | WB | 74 | 24.3 |
| Yin, 2005^81^ | - | China | Outpatient clinic | CS | Conv | STI clinic attendees | WB | 105 | 55.2 |
| **Other populations** | | | | | | | | | |
| Nakagawa, 1991^33^ | - | Japan | Hospital | CS | Conv | Patients with malignancies | Immunodot | 15 | 40.0 |
| Peng, 1991^34^ | 1987-88 | China | Outpatient clinic | CC | Conv | Women with invasive cervical cancer | WB | 89 | 42.0 |
| Wang, 2014^82^ | 2009 | South Korea | Outpatient clinic | CS | RS | Males with anal disease | ELISA | 2,038 | 24.0 |

^a^ The reported study design is the original study design (case control, cross sectional, cohort, or randomized controlled trial). The included seroprevalence measures are those for the baseline measures at the beginning of the study.

Abbreviations: CC = Case-control, Conv = Convenience, CRS = Cluster random sampling, CS = Cross-sectional, CSWs = Commercial sex workers, ELISA = Enzyme-linked immunosorbent type-specific assay, FSWs = Female sex workers, HIV = Human immunodeficiency virus, HSV-2 = Herpes simplex virus type 2, MSCS = Multiple stage cluster sampling, MSM = Men who have sex with men, RDS = Respondent driven sampling, RS = Random sampling, SRS = Stratified random sampling, SS = Snowball sampling, STI = Sexually transmitted infection, TL & SS = Time location and snowball sampling, VCT = Voluntary counselling and testing, WB = Western blot.

# **Supplementary Table S5.** Studies reporting HSV-2 seroprevalence in South Asia.

| **Author, year** | **Year(s) of data collection** | **Country** | **Study site** | **Original study design^a^** | **Sampling method** | **Population** | **HSV-2 serological assay** | **Sample size** | **HSV-2 seroprevalence (%)** |
| --- | --- | --- | --- | --- | --- | --- | --- | --- | --- |
| **General populations** | | | | | | | | | |
| Adamson, 2011^83^ | 2005-06 | India | Outpatient clinic | CS | Conv | 15-30 years old sexually active women | ELISA | 897 | 11.5 |
| Banandur, 2011^84^ | 2006-08 | India | Community | CS | CRS | 15-49 years old general population | ELISA | 1,413 | 13.2 |
| Becker, 2007^85^ | 2003 | India | Community | CS | RS | General population in Karnataka | ELISA | 901 | 18.9 |
| Biswas, 2011^86^ | 2007-09 | India | Outpatient clinic | CS | Conv | Pregnant women | ELISA | 1,640 | 8.7 |
| Bochner, 2013^87^ | 2009 | India | Outpatient clinic | CS | Conv | >15 years old pregnant women | ELISA | 478 | 6.6 |
| Bogaerts, 2001^4^ | 1996-98 | Bangladesh | Outpatient clinic | CS | Conv | 1-12 years old children | ELISA | 79 | 2.5 |
| Bogaerts, 2001^4^ | 1996-98 | Bangladesh | Outpatient clinic | CS | Conv | Married and unmarried women | ELISA | 2,343 | 11.7 |
| Chawla, 2008^88^ | - | India | Community | CS | CRS | Females in urban communities in Delhi | WB | 116 | 8.6 |
| Chawla, 2008^88^ | - | India | Community | CS | CRS | Males in urban communities in Delhi | WB | 85 | 7.0 |
| Coudray, 2019^89^ | 2016-17 | India | Community | CS | Conv | Healthy population | ELISA | 351 | 9.4 |
| Cowan, 2003^90^ | 2000 | Sri Lanka | Outpatient clinic | CS | Conv | Healthy men | ELISA | 1,619 | 14.1 |
| Cowan, 2003^90^ | 2000 | Sri Lanka | Outpatient clinic | CS | Conv | Health women | ELISA | 684 | 21.2 |
| Cowan, 2003^90^ | 2000 | Sri Lanka | Outpatient clinic | CS | Conv | Antenatal clinic attendees | ELISA | 757 | 8.3 |
| Cowan, 2003^90^ | 2000 | Sri Lanka | Outpatient clinic | CS | Conv | 1-11 years old children | ELISA | 433 | 4.8 |
| Cowan, 2003^90^ | 2000 | India | Outpatient clinic | CS | Conv | <15 years old individuals in India | ELISA | 361 | 2.2 |
| Cowan, 2003^90^ | 2000 | India | Outpatient clinic | CS | Conv | Antenatal clinic attendees | ELISA | 990 | 7.9 |
| Cowan, 2003^90^ | 2000 | India | Outpatient clinic | CS | Conv | Male blood donors | ELISA | 394 | 10.2 |
| Cowan, 2003^90^ | 2000 | India | Outpatient clinic | CS | Conv | Female blood donors | ELISA | 48 | 14.6 |
| Das, 1998^91^ | - | India | Outpatient clinic | CC | Conv | Women attending antenatal clinics | EIA | 150 | 26.0 |
| Hochberg, 2015^5^ | 2004-05 | India | Community | Cohort | SRS | Healthy adults | ELISA | 8,494 | 7.5 |
| Madhivanan, 2011^6^ | 2005-06 | India | Outpatient clinic | Cohort | Conv | 15-30 years old sexually active women | ELISA | 882 | 12.1 |
| Munawwar, 2018^92^ | 2010-13 | India | Outpatient clinic | CC | Conv | Healthy males | ELISA | 118 | 5.1 |
| Munro, 2008^93^ | 2005-06 | India | Community | CS | CRS | Mysore population | ELISA | 4,199 | 11.6 |
| Nag, 2015^94^ | 2012-13 | India | Outpatient clinic | CS | Conv | Healthy adults | ELISA | 45 | 17.8 |
| Nasrallah, 2018^95^ | 2013-16 | India | Hospital | CS | RS | >18 years old male blood donors | ELISA | 325 | 3.7 |
| Panchanadeswara, 2006^96^ | 2001 | India | Community | CS | RS | >18 years old men and women | ELISA | 1,620 | 13.2 |
| Raj, 2011^97^ | 2008-09 | India | Outpatient clinic | CS | Conv | Healthy controls | ELISA | 35 | 0.0 |
| Rajaram, 2011^98^ | 2003 | India | Community | CS | CRS | Healthy population in the first survey | ELISA | 901 | 22.1 |
| Rajaram, 2011^98^ | 2009-10 | India | Community | CS | CRS | Healthy population in the second survey | ELISA | 550 | 20.3 |
| Rathore, 2010^99^ | 2008-09 | India | Outpatient clinic | CS | RS | 16-40 years old pregnant women | ELISA | 200 | 7.5 |
| Schensul, 2007^100^ | 2003 | India | Community | CS | CRS | Married men in Mumbai | ELISA | 641 | 9.7 |
| Schneider, 2010b^101^ | 2004-05 | India | Community | CS | SRS | 15-49 years old men and women | ELISA | 12,617 | 5.9 |
| Sgaier, 2011^102^ | 2006 | India | Community | CS | CRS | >18 years old from the general population | ELISA | 2,347 | 10.1 |
| **Intermediate-risk populations** | | | | | | | | | |
| Celentano, 2010^103^ | 2001-04 | India | Community | CS | CRS | Women working in entertainment establishments | ELISA | 607 | 56.8 |
| Celentano, 2010^103^ | 2001-04 | India | Community | CS | CRS | Men working in entertainment establishments | ELISA | 2,913 | 8.6 |
| Gibney, 2001^104^ | 1998 | Bangladesh | Community | CS | RS | Women living in slum areas | ELISA | 384 | 32.0 |
| Gibney, 2002^105^ | 1998-99 | Bangladesh | Community | CS | RS | Truck drivers and helpers | ELISA | 387 | 25.8 |
| Go, 2007^106^ | 2001-02 | India | Community | CS | RS | Alcohol venue male patrons | ELISA | 654 | 17.1 |
| Go, 2007^106^ | 2001-02 | India | Community | CS | RS | Household sample of men living in a slum | ELISA | 685 | 9.9 |
| Haseen, 2012^107^ | 2006-07 | Bangladesh | Community | CS | CRS | Clients of FSWs | ELISA | 994 | 12.9 |
| National Summary Report^108^ | 2007 | India | Community | CS | TLS | PWID in Maharashtra | ELISA | 71 | 32.4 |
| National Summary Report^108^ | 2006 | India | Community | CS | TLS | PWID in Manipur | ELISA | 166 | 13.9 |
| National Summary Report^108^ | 2006 | India | Community | CS | TLS | PWID in Nagaland | ELISA | 169 | 13.6 |
| National Summary Report^108^ | 2006-07 | India | Community | CS | TLS | Clients of FSWs in Andhra Pradesh | ELISA | 404 | 42.3 |
| National Summary Report^108^ | 2007 | India | Community | CS | TLS | Clients of FSWs in Maharashtra | ELISA | 317 | 21.8 |
| National Summary Report^108^ | 2006-07 | India | Community | CS | TLS | Clients of FSWs in Tamilnandu | ELISA | 242 | 20.3 |
| National Summary Report^108^ | 2006-07 | India | Community | CS | TLS | Truck drivers | ELISA | 415 | 24.8 |
| Panda, 2007^109^ | 2003 | India | Community | CS | Conv | 18-45 years old male PWID | ELISA | 211 | 40.0 |
| Panda, 2007^109^ | 2003 | India | Community | CS | Conv | 18-45 years old females who are sexual partners of PWID | ELISA | 211 | 38.0 |
| Panda, 2014^110^ | 2010 | India | Community | CS | RS | >18 years old males who inject drugs | ELISA | 1,155 | 10.0 |
| Schneider, 2010a^101^ | 2007-09 | India | Community | CS | Conv | Truck drivers in Hyderabad | ELISA | 90 | 5.6 |
| Shaw, 2011b^111^ | 2008 | India | Community | CS | MSCS | Clients of FSWs | EIA | 2,610 | 28.0 |
| Uma, 2006^112^ | 2002 | India | Community | CS | Conv | Women living in slum areas | ELISA | 487 | 15.6 |
| **Higher-risk populations** | | | | | | | | | |
| Barua, 2012^113^ | 2006 | India | Community | CS | RDS | FSWs | ELISA | 426 | 37.8 |
| Gutierrez, 2010^114^ | 2003-04 | India | Community | CS | RDS | MSM at the non-FPP sub-sites during the first survey | ELISA | 1,106 | 34.0 |
| Gutierrez, 2010^114^ | 2003-04 | India | Community | CS | RDS | MSM at the FPP sub-sites during the first survey | ELISA | 1,680 | 40.0 |
| Gutierrez, 2010^114^ | 2007 | India | Community | CS | RDS | MSM at the non-FPP sub-sites during the second survey | ELISA | 218 | 29.0 |
| Gutierrez, 2010^114^ | 2007 | India | Community | CS | RDS | MSM at the FPP sub-sites during the second survey | ELISA | 1,317 | 32.0 |
| Gutierrez, 2010^114^ | 2003-04 | India | Community | CS | RDS | FSWs at the non-FPP sub-sites during the first survey | ELISA | 1,750 | 46.0 |
| Gutierrez, 2010^114^ | 2003-04 | India | Community | CS | RDS | FSWs at the FPP sub-sites during the first survey | ELISA | 1,692 | 47.0 |
| Gutierrez, 2010^114^ | 2007 | India | Community | CS | RDS | FSWs at the non-FPP sub-sites during the second survey | ELISA | 855 | 21.0 |
| Gutierrez, 2010^114^ | 2007 | India | Community | CS | RDS | FSWs at the FPP sub-sites during the second survey | ELISA | 1,292 | 29.0 |
| Mishra, 2009^115^ | 2004-06 | India | Community | CS | RS | <30 years old FSWs in Karnataka | ELISA | 997 | 57.9 |
| Mishra, 2009^115^ | 2004-06 | India | Community | CS | RS | >30 years old FSWs in Karnataka | ELISA | 1,211 | 75.8 |
| National Summary Report^108^ | 2005-09 | India | Community | CS | MSCS | FSWs in Andhra Pradesh | ELISA | 650 | 81.9 |
| National Summary Report^108^ | 2005-09 | India | Community | CS | MSCS | FSWs in Karnataka | ELISA | 184 | 70.1 |
| National Summary Report^108^ | 2005-09 | India | Community | CS | MSCS | FSWs in Maharashtra | ELISA | 653 | 72.6 |
| National Summary Report^108^ | 2005-09 | India | Community | CS | MSCS | FSWs in Nagaland | ELISA | 84 | 48.8 |
| National Summary Report^108^ | 2005-09 | India | Community | CS | MSCS | FSWs in Tamil Nadu | ELISA | 405 | 55.8 |
| National Summary Report^108^ | 2006 | India | Community | CS | MSCS | MSM in Andhra Pradesh | ELISA | 322 | 53.4 |
| National Summary Report^108^ | 2006 | India | Community | CS | MSCS | MSM in Maharashta | ELISA | 130 | 33.9 |
| National Summary Report^108^ | 2006 | India | Community | CS | MSCS | MSM in Tamil Nadu | ELISA | 324 | 28.7 |
| National Summary Report^108^ | 2006 | India | Community | CS | MSCS | MSM in Kartanaka | ELISA | 30 | 36.7 |
| National Summary Report^108^ | 2006 | India | Community | CS | MSCS | Transgender in Tamil Nandu | ELISA | 80 | 45.0 |
| Nessa, 2004^116^ | 2002 | Bangladesh | Community | CS | CRS | Hotel-based FSWs | ELISA | 400 | 34.5 |
| Qutub, 2003^117^ | - | Bangladesh | Community | CS | Conv | FSWs | ELISA | 463 | 94.6 |
| Rahman, 2000^118^ | 1998 | Bangladesh | Community | CS | Conv | FSWs | ELISA | 203 | 62.5 |
| Reza-Paul, 2008^119^ | 2004 | India | Community | CS | TLS | FSWs in Mysore in 2004 | ELISA | 393 | 64.4 |
| Reza-Paul, 2008^119^ | 2006 | India | Community | CS | TLS | FSWs in Mysore in 2006 | ELISA | 425 | 79.0 |
| Sarna, 2013^120^ | 2010 | India | Outpatient clinic | CS | RDS | FSWs in Nellore | ELISA | 529 | 60.7 |
| Setia, 2006^121^ | - | India | Outpatient clinic | CS | Conv | >18 years old MSM attending STI clinic | ELISA | 121 | 40.0 |
| Setia, 2006^121^ | - | India | Outpatient clinic | CS | Conv | >18 years old transgender attending STI clinic | ELISA | 28 | 71.0 |
| Shahmanesh, 2009^122^ | 2004-05 | India | Community | CS | RDS | FSWs | ELISA | 326 | 57.2 |
| Solomon, 2010^123^ | 2008 | India | Community | CS | RDS | MSM in Tamil Nandu | ELISA | 721 | 25.8 |
| Solomon, 2015^124^ | 2012-13 | India | Community | CS | RDS | MSM in India | ELISA | 11,997 | 18.9 |
| Uma, 2005^125^ | 2004 | India | Community | CS | Conv | FSWs with positive bacterial vaginosis | ELISA | 260 | 73.4 |
| Uma, 2005^125^ | 2004 | India | Community | CS | Conv | FSWs with intermediate bacterial vaginosis | ELISA | 92 | 67.3 |
| Uma, 2005^125^ | 2004 | India | Community | CS | Conv | FSWs with negative bacterial vaginosis | ELISA | 230 | 56.0 |
| **HIV-positive individuals and individuals in HIV-discordant couples** | | | | | | | | | |
| Munawwar, 2018^92^ | 2010-13 | India | Outpatient clinic | CC | Conv | HIV-positive males | ELISA | 233 | 39.9 |
| Nag, 2015^94^ | 2012-13 | India | Outpatient clinic | CS | Conv | HIV-positive patients | ELISA | 52 | 61.5 |
| Nag, 2015^94^ | 2012-13 | India | Outpatient clinic | CS | Conv | HIV-positive patients | ELISA | 45 | 57.8 |
| Venkatesh, 2011^126^ | 2008 | India | Outpatient clinic | CS | Conv | HIV-positive patients | ELISA | 144 | 24.3 |
| Venkatesh, 2011^126^ | 2008 | India | Outpatient clinic | CS | Conv | Partners of HIV-positive patients | ELISA | 103 | 15.5 |
| **STI clinic attendees and symptomatic populations** | | | | | | | | | |
| Becker, 2010^127^ | 2004-06 | India | Outpatient clinic | CS | Conv | Males with urethral discharge | ELISA | 161 | 31.7 |
| Becker, 2010^127^ | 2004-06 | India | Outpatient clinic | CS | Conv | Females with vaginal discharge | ELISA | 81 | 18.5 |
| Becker, 2010^127^ | 2004-06 | India | Outpatient clinic | CS | Conv | Females with vaginal discharge and clinical cervicitis | ELISA | 263 | 24.3 |
| Becker, 2010^127^ | 2004 | India | Outpatient clinic | CS | Conv | Males with non-herpetic GUD | ELISA | 190 | 49.5 |
| Becker, 2010^127^ | 2004 | India | Outpatient clinic | CS | Conv | Females with non-herpetic GUD | ELISA | 23 | 60.9 |
| Becker, 2010^127^ | 2004 | India | Outpatient clinic | CS | Conv | Males with herpetic GUD | ELISA | 18 | 33.3 |
| Becker, 2010^127^ | 2004 | India | Outpatient clinic | CS | Conv | Females with herpetic GUD | ELISA | 45 | 68.9 |
| Cowan, 2003^90^ | 2000 | Sri Lanka | Outpatient clinic | CS | Conv | Females attending an STI clinic | ELISA | 276 | 49.3 |
| Cowan, 2003^90^ | 2000 | Sri Lanka | Outpatient clinic | CS | Conv | Males attending an STI clinic | ELISA | 400 | 33.0 |
| Das, 1998^91^ | - | India | Outpatient clinic | CS | Conv | High-risk cases including patients attending STI clinic | EIA | 200 | 72.0 |
| Kumarasamy, 2008^13^ | 2002 | India | Outpatient clinic | CS | Conv | >18 years old men | ELISA | 236 | 29.0 |
| Kumarasamy, 2008^13^ | 2002 | India | Outpatient clinic | CS | Conv | >18 years old women | ELISA | 244 | 50.0 |
| Patwardhan, 2016^128^ | - | India | Outpatient clinic | CS | Conv | Patients with primary genital herpes | ELISA | 21 | 14.0 |
| Patwardhan, 2016^128^ | - | India | Outpatient clinic | CS | Conv | Patients with recurrent genital herpes | ELISA | 23 | 83.0 |
| Raj, 2011^97^ | 2008-09 | India | Outpatient clinic | CS | Conv | Patients with symptoms of viral STI | ELISA | 203 | 29.6 |
| Reynolds, 2003^14^ | 1993-00 | India | Outpatient clinic | CS | Conv | Mixed at-risk populations^b^ | ELISA | 2,732 | 43.0 |
| Shivanand, 2019^129^ | - | India | Outpatient clinic | CS | Conv | Patients with genital herpes | ELISA | 70 | 85.7 |

^a^ The reported study design is the original study design (case control, cross sectional, cohort, or randomized controlled trial). The included seroprevalence measures are those for the baseline measures at the beginning of the study.

^b^ Mixed population includes male patients with STIs, female partners of male patients with STIs, FSWs, and women with reproductive tract infections

Abbreviations: CC = Case-control, Conv = Convenience, CS = Cross-sectional, CRS = Cluster random sampling, EIA = Enzyme immunoassay, ELISA = Enzyme-linked immunosorbent type-specific assay, FPP = Frontiers prevention project, FSWs = Female sex workers, GUD = Genital ulcer disease, HIV = Human immunodeficiency virus, HSV-2 = Herpes simplex virus type 2, LSS = low socioeconomic status, MSCS = Multiple stage cluster sampling, MSM = Men who have sex with men, Non-FPP = Non-frontiers prevention project, PWID = People who inject drugs, RDS = Respondent driven sampling, RS = Random sampling, SRS = Stratified random sampling, STI = Sexually transmitted infection, TLS = Time-location sampling, WB = Western blot.

# **Supplementary Table S6.** Studies reporting HSV-2 seroprevalence in Southeast Asia among different populations.

| **Author, year** | **Year(s) of data collection** | **Country** | **Study site** | **Original study design^a^** | **Sampling method** | **Population** | **HSV-2 serological assay** | **Sample size** | **HSV-2 seroprevalence (%)** |
| --- | --- | --- | --- | --- | --- | --- | --- | --- | --- |
| **General populations** | | | | | | | | | |
| Ashley-Morrow, 2004^17^ | 2000-01 | Thailand | Community | CS | Conv | Adult women | WB | 98 | 44.9 |
| Davies, 2007^130^ | 1999-00 | Indonesia | Outpatient clinic | CS | Conv | Women attending maternal and child health clinic in Bali | ELISA | 228 | 21.9 |
| Davies, 2007^130^ | 1999-00 | Indonesia | Outpatient clinic | CS | Conv | Pregnant women attending two antenatal clinics | ELISA | 47 | 12.8 |
| Davies, 2007^130^ | 1999-00 | Indonesia | Outpatient clinic | CS | Conv | Women attending two gynecology clinics in Makassar | ELISA | 61 | 14.8 |
| Joesoef, 1996^131^ | 1992-93 | Indonesia | Outpatient clinic | CS | Conv | Pregnant women | Immunodot | 599 | 9.9 |
| Keorochana, 2018^132^ | 2017 | Thailand | Hospital | CS | Conv | Patients undergoing elective cataract surgery | ELISA | 84 | 83.3 |
| Le, 2009^133^ | 1997 | Vietnam | Outpatient clinic | CS | RS | 15-69 years old women living in Ho Chi Minh | ELISA | 1,106 | 34.4 |
| Le, 2009^133^ | 1997 | Vietnam | Outpatient clinic | CS | RS | 15-69 years old women living in Hanoi | ELISA | 1,170 | 9.2 |
| Nasrallah, 2018^95^ | 2013-16 | Philippines | Hospital | CS | Conv | Male blood donors | ELISA | 120 | 8.3 |
| Ngo, 2008b^134^ | 2004 | Vietnam | Community | CS | Conv | Married women | ELISA | 1,238 | 8.7 |
| Smith, 2001^135^ | 1991-93 | Philippines | Outpatient clinic | CS | Conv | Healthy population | ELISA | 371 | 9.2 |
| Smith, 2002^136^ | 1990-93 | Thailand | Outpatient clinic | CC | Conv | Control women | WB | 75 | 34.7 |
| Sukvirach, 2003^137^ | 1997-00 | Thailand | Community | CS | RS | Human papillomavirus uninfected women | ELISA | 1,631 | 28.9 |
| Van de Wijgert, 2008^138^ | 1999-02 | Thailand | Outpatient clinic | RCT | RS | Women receiving Carraguard® gel | ELISA | 83 | 42.2 |
| Van de Wijgert, 2008^138^ | 1999-02 | Thailand | Outpatient clinic | RCT | RS | Women receiving placebo gel | ELISA | 82 | 58.5 |
| **Intermediate-risk populations** | | | | | | | | | |
| Celentano, 2008^139^ | 2005-06 | Thailand | Community | CS | RDS | Young methamphetamine users | ELISA | 658 | 8.0 |
| Dobbins, 1999^140^ | 1991 | Thailand | Community | CS | RS | Male Thai army conscripts who entered military service | Immunodot | 1,115 | 14.9 |
| Go, 2006^141^ | 2003 | Vietnam | Community | CS | SS | PWID | ELISA | 272 | 22.4 |
| Nguyen, 2009^142^ | 2007 | Vietnam | Community | CS | TLS | Active bridgers | ELISA | 16 | 33.3 |
| Nguyen, 2009^142^ | 2007 | Vietnam | Community | CS | TLS | Potential bridgers | ELISA | 147 | 21.2 |
| Nguyen, 2009^142^ | 2007 | Vietnam | Community | CS | TLS | Unlikely bridgers | ELISA | 129 | 8.8 |
| Pisani, 2006^143^ | 2003 | Timor-Leste | Community | CS | Conv | Male taxi drivers | ELISA | 207 | 29.0 |
| Pisani, 2006^143^ | 2003 | Timor-Leste | Community | CS | Conv | Male soldiers | ELISA | 248 | 11.7 |
| **Higher-risk populations** | | | | | | | | | |
| Davies, 2007^130^ | 1999-00 | Indonesia | Outpatient clinic | CS | Conv | FSWs attending two STI clinics in Kupang | ELISA | 165 | 90.3 |
| Limpakarnjanarat, 1999^144^ | 1991-94 | Thailand | Outpatient clinic | CS | Conv | FSWs work in brothels and non-brothels venues | Immunodot | 500 | 75.6 |
| Pisani, 2006^143^ | 2003 | Timor-Leste | Community | CS | Conv | MSM | ELISA | 110 | 29.1 |
| Pisani, 2006^143^ | 2003 | Timor-Leste | Community | CS | Conv | FSWs | ELISA | 98 | 60.2 |
| Theng, 2006b^145^ | 2003-04 | Singapore | Outpatient clinic | CS | RS | FSWs | ELISA | 300 | 79.0 |
| Van Griensven, 2013^146^ | 2006-10 | Thailand | Outpatient clinic | Cohort | Conv | Adult MSM | ELISA | 1,740 | 21.3 |
| Vu, 2007^147^ | 2002 | Vietnam | Community | CS | Conv | FSWs in five bordering provinces in 2002 | ELISA | 903 | 27.7 |
| Vu, 2007^147^ | 2004 | Vietnam | Community | CS | Conv | FSWs in five bordering provinces in 2004 | ELISA | 979 | 24.9 |
| **HIV-positive individuals and individuals in HIV-discordant couples** | | | | | | | | | |
| Bollen, 2008^11^ | 1996-97 | Thailand | Outpatient clinic | RCT | Conv | HIV-positive pregnant women | ELISA | 307 | 74.3 |
| Chu, 2006^148^ | 2000-01 | Thailand | Community | CS | Conv | HIV-positive females | ELISA | 71 | 80.0 |
| Chu, 2006^148^ | 2000-01 | Thailand | Community | CS | Conv | HIV-positive males | ELISA | 69 | 68.0 |
| Yap, 2017^149^ | - | Malaysia | Hospital | CS | Conv | HIV-positive patients | ELISA | 232 | 53.9 |
| **STI clinic attendees and symptomatic populations** | | | | | | | | | |
| Davies, 2007^130^ | 1999-00 | Indonesia | Outpatient clinic | CS | Conv | Men attending two STI clinics in Bali | ELISA | 96 | 24.0 |
| Davies, 2007^130^ | 1999-00 | Indonesia | Outpatient clinic | CS | Conv | Women attending two STI clinics in Bali | ELISA | 66 | 19.7 |
| Davies, 2007^130^ | 1999-00 | Indonesia | Outpatient clinic | CS | Conv | Men attending two STI clinic in Makssar | ELISA | 20 | 10.0 |
| Davies, 2007^130^ | 1999-00 | Indonesia | Outpatient clinic | CS | Conv | Women attending two STI clinics in Makssar | ELISA | 27 | 14.8 |
| Theng, 2006a^150^ | 2003-04 | Singapore | Outpatient clinic | CS | RS | STI clinic attendees | ELISA | 400 | 28.5 |
| **Other populations** | | | | | | | | | |
| Smith, 2002^136^ | 1990-93 | Thailand | Outpatient clinic | CC | Conv | Women with adeno- or adenosquamous carcinoma | WB | 21 | 61.9 |
| Smith, 2002^136^ | 1990-93 | Thailand | Outpatient clinic | CC | Conv | Women with squamous-cell carcinoma | WB | 202 | 59.4 |
| Smith, 2002^136^ | 1991-93 | Philippines | Outpatient clinic | CC | Conv | Women with squamous-cell carcinoma | ELISA | 321 | 30.8 |
| Smith, 2002^136^ | 1991-93 | Philippines | Outpatient clinic | CC | Conv | Women with adeno- or adenosquamous carcinoma | ELISA | 33 | 15.2 |
| Sukvirach, 2003^137^ | 1997-00 | Thailand | Community | CS | RS | Human papillomavirus infected women | ELISA | 110 | 48.2 |

^a^ The reported study design is the original study design (case control, cross sectional, cohort, or randomized controlled trial). The included seroprevalence measures are those for the baseline measures at the beginning of the study.

Abbreviations: CC = Case-control, Conv = Convenience, CS = Cross-sectional, ELISA = Enzyme-linked immunosorbent type-specific assay, FSWs = Female sex workers, HIV = Human immunodeficiency virus, HSV-2 = Herpes simplex virus type 2, MSM = Men who have sex with men, RCT = Randomized controlled trial, RDS = Respondent driven sampling, RS = Random sampling, SS = Snowball sampling, STI = Sexually transmitted infection, TLS = Time location sampling, WB = Western blot.

# **Supplementary Table S7.** Studies reporting HSV-2 seroprevalence in Papua New Guinea.

| **Author, year** | **Year(s) of data collection** | **Country** | **Study site** | **Original study design^a^** | **Sampling method** | **Population** | **HSV-2 serological assay** | **Sample size** | **HSV-2 seroprevalence (%)** |
| --- | --- | --- | --- | --- | --- | --- | --- | --- | --- |
| **General populations** | | |  |  |  |  |  |  |  |
| Rezza, 2001^151^ | 1999-99 | Papua New Guinea | Community | CS | Conv | General population in Bensbach | ELISA | 54 | 29.6 |
| Rezza, 2001^151^ | 1999-99 | Papua New Guinea | Community | CS | Conv | General population in Port Moresby | ELISA | 93 | 7.5 |
| Suligoi, 2005^152^ | 2001-01 | Papua New Guinea | Community | CS | Conv | Melanesians residing in six remote villages | ELISA | 343 | 27.4 |
| Vallely, 2016^153^ | - | Papua New Guinea | Outpatient clinic | CS | Conv | Women attending antenatal clinics | ELISA | 765 | 28.0 |
| Vallely, 2017^154^ | 2013-15 | Papua New Guinea | VCT | CS | Conv | Men attending a VCT clinic | ELISA | 1,073 | 33.6 |
| **STI clinic attendees and symptomatic population** | | | | | | | | | |
| Ryan, 2014^155^ | 2010-12 | Papua New Guinea | Outpatient clinic | Cohort | Conv | STI clinic attendees | ELISA | 132 | 60.6 |
| Valelly, 2014^16^ | 2011-12 | Papua New Guinea | Outpatient clinic | Cohort | Conv | Patients attending a sexual health clinic | ELISA | 154 | 46.1 |

^a^ The reported study design is the original study design (case control, cross sectional, cohort, or randomized controlled trial). The included seroprevalence measures are those for the baseline measures at the beginning of the study.

Abbreviations: Conv = Convenience, CS = Cross-sectional, ELISA = Enzyme-linked immunosorbent type-specific assay, HSV-2 = Herpes simplex virus type 2, VCT = Voluntary counselling and testing, STI = Sexually transmitted infection.

# **Supplementary Figure S1.** Forest plots presenting outcomes of the pooled mean HSV-2 seroprevalence among different populations in Asia.

##

General populations











##

Intermediate-risk populations

##

Higher-risk populations








## STI clinic attendees and symptomatic populations





##

HIV-positive individuals and individuals in HIV-discordant couples

## Other populations





# **Supplementary Table S8.** Univariable and multivariable meta-regression analyses for HSV-2 seroprevalence in Asia using the year of publication instead of the year of data collection as the time variable.

|  | | | **Outcome measures** | **Sample size** | **Univariable analysis** | | | | **Multivariable analysis** | | | |
| --- | --- | --- | --- | --- | --- | --- | --- | --- | --- | --- | --- | --- |
|  | | | **Total n** | **Total N** | ***RR* (95%CI)** | **p-value** | **LR test p-value** | **Adjusted R^2^ (%)** | **Model 3^a^** | | **Model 4^b^** | |
|  | | |  |  |  |  |  |  | ***ARR* (95% CI)** | **p-value** | ***ARR* (95% CI)** | **p-value** |
| **Population characteristics** | **Population type** | General populations | 295 | 104,188 | 1.00 | - | <0.001 | 40.30 | 1.00 | - | 1.00 | - |
|  |  | Intermediate-risk populations | 86 | 23,946 | 1.58 (1.32-1.85) | <0.001 |  |  | 1.79 (1.49-2.14) | <0.001 | 1.82 (1.52-2.18) | <0.001 |
|  |  | Higher-risk populations | 209 | 73,139 | 3.40 (2.99-3.87) | <0.001 |  |  | 3.48 (3.06-3.95) | <0.001 | 3.47 (3.05-3.94) | <0.001 |
|  |  | STI clinic attendees and symptomatic populations | 46 | 6,610 | 3.19 (2.54-3.99) | <0.001 |  |  | 2.38 (1.96-2.89) | <0.001 | 2.35 (1.93-2.86) | <0.001 |
|  |  | HIV-positive individuals and individuals in HIV-discordant couples | 29 | 5,476 | 3.63 (2.77-4.75) | <0.001 |  |  | 3.69 (2.92-4.66) | <0.001 | 3.60 (2.84-4.56) | <0.001 |
|  |  | Other populations^c^ | 13 | 2,799 | 2.34 (1.56-3.49) | <0.001 |  |  | 2.00 (1.45-2.76) | <0.001 | 2.11 (1.52-2.92) | <0.001 |
|  | **Age group** | <20 years | 21 | 4,257 | 1.00 | - | <0.001 | 16.01 | 1.00 | - | 1.00 | - |
|  |  | 20-29 years | 63 | 16,222 | 1.10 (0.69-1.73) | 0.695 |  |  | 1.20 (0.87-1.65) | 0.237 | 1.18 (0.85-1.63) | 0.331 |
|  |  | 30-39 years | 57 | 12,163 | 1.32 (0.83-2.10) | 0.236 |  |  | 1.65 (1.19-2.29) | 0.002 | 1.60 (1.15-2.23) | 0.005 |
|  |  | 40-49 years | 40 | 9,111 | 1.45 (0.89-2.36) | 0.131 |  |  | 1.91 (1.36-2.70) | <0.001 | 1.85 (1.31-2.62) | 0.001 |
|  |  | 50-59 years | 14 | 1,620 | 1.76 (0.96-3.24) | 0.067 |  |  | 2.70 (1.74-4.18) | <0.001 | 2.75 (1.77-4.27) | <0.001 |
|  |  | ≥60 years | 14 | 1,326 | 2.77 (1.52-5.04) | 0.001 |  |  | 4.15 (2.71-6.37) | <0.001 | 4.22 (2.74-6.52) | <0.001 |
|  |  | Mixed | 469 | 171,459 | 2.93 (1.95-4.40) | <0.001 |  |  | 1.76 (1.32-2.36) | <0.001 | 1.71 (1.28-2.30) | <0.001 |
|  | **Sex** | Women | 342 | 115,383 | 1.00 | - | <0.001 | 8.24 | 1.00 | - | 1.00 | - |
|  |  | Men | 306 | 91,939 | 0.61 (0.53-0.70) | <0.001 |  |  | 0.60 (0.54-0.67) | <0.001 | 0.59 (0.53-0.65) | <0.001 |
|  |  | Mixed sexes | 30 | 8,836 | 1.14 (0.82-1.58) | 0.437 |  |  | 1.25 (1.00-1.57) | 0.049 | 1.19 (0.95-1.49) | 0.131 |
|  | **Subregions** | East Asia | 268 | 100,501 | 1.00 | - | <0.001 | 3.10 | 1.00 | - | 1.00 | - |
|  |  | South Asia | 309 | 96,357 | 1.42 (1.22-1.65) | <0.001 |  |  | 2.17 (1.62-2.90) | <0.001 | 2.27 (1.70-3.04) | <0.001 |
|  |  | Southeast Asia | 83 | 16,554 | 1.45 (1.16-1.82) | 0.001 |  |  | 1.57 (1.28-1.92) | <0.001 | 1.52 (1.23-1.87) | <0.001 |
|  |  | Papua New Guinea | 18 | 2,746 | 1.82 (1.18-2.81) | 0.006 |  |  | 4.04 (2.76-5.93) | <0.001 | 4.23 (2.87-6.24) | <0.001 |
|  | **National income** | LMIC | 369 | 106,572 | 1.00 | - | 0.004 | 1.32 | 1.00 | - | 1.00 | - |
|  |  | UMIC | 185 | 97,549 | 0.76 (0.65-0.90) | 0.001 |  |  | 1.60 (1.23-2.08) | <0.001 | 1.61 (1.24-2.10) | <0.001 |
|  |  | HIC | 124 | 12,037 | 0.87 (0.71-1.06) | 0.167 |  |  | 2.23 (1.67-3.00) | <0.001 | 2.27 (1.69-3.06) | <0.001 |
| **Study methodology characteristics** | **Assay type** | Western Blot | 44 | 4,884 | 1.00 | - | 0.200 | 0.23 | - | - | - | - |
|  |  | ELISA | 634 | 211,274 | 0.82 (0.61-1.11) | 0.200 |  |  | - | - | - | - |
|  | **Sample size**^d^ | <200 | 185 | 9,414 | 1.00 | - | <0.001 | 14.57 | 1.00 | - | 1.00 | - |
|  |  | ≥200 | 493 | 206,744 | 0.48 (0.41-0.56) | <0.001 |  |  | 0.79 (0.69-0.90) | <0.001 | 0.78 (0.68-0.89) | <0.001 |
|  | **Sampling method** | Probability-based | 298 | 100,245 | 1.00 | - | 0.032 | 0.60 | 1.00 | - | 1.00 | - |
|  |  | Non-probability-based | 380 | 115,913 | 1.17 (1.01-1.35) | 0.032 |  |  | 1.21 (1.07-1.36) | 0.003 | 1.17 (1.04-1.33) | 0.010 |
|  | **Response rate** | ≥80% | 129 | 53,942 | 1.00 | - | <0.001 | 8.83 | 1.00 | - | 1.00 | - |
|  |  | <80% | 165 | 28,416 | 2.19 (1.79-2.69) | <0.001 |  |  | 0.95 (0.81-1.12) | 0.559 | 0.99 (0.85-1.17) | 0.947 |
|  |  | Unclear | 384 | 133,800 | 1.50 (1.25-1.79) | <0.001 |  |  | 0.86 (0.75-0.99) | 0.037 | 0.92 (0.80-1.05) | 0.212 |
| **Temporal variables** | **Year of publication category** | ≤2005 | 136 | 23,075 | 1.00 | - | <0.001 | 4.32 | 1.00 | - | - | - |
|  |  | 2006-2015 | 469 | 139,567 | 1.02 (0.85-1.22) | 0.856 |  |  | 0.79 (0.69-0.91) | 0.001 | - | - |
|  |  | >2015 | 73 | 53,516 | 0.56 (0.43-0.73) | <0.001 |  |  | 0.54 (0.44-0.65) | <0.001 | - | - |
|  | **Year of publication** | | 678 | 216,158 | 0.98 (0.97-1.00) | 0.024 | 0.024 | 1.01 | - | - | 0.98 (0.97-0.99) | <0.001 |

^a^ Variance explained by multivariable model 3 (adjusted *R^2^*) = 65.98%.

^b^ Variance explained by multivariable model 4 (adjusted *R^2^*) = 64.79%.

^c^ Other populations include populations with an undetermined risk of acquiring HSV-2 infection such as patients with cervical cancer.

^d^ Sample size denotes the sample size of each study population found in the original publication.

Abbreviations: ARR = Adjusted risk ratio, CI = Confidence interval*,* ELISA = Enzyme-linked immunosorbent type-specific assay, HIC = High-income country, HIV = human immunodeficiency virus, HSV-2 = Herpes simplex virus type 2, LMIC = Lower-middle-income country, LR = Likelihood ratio, RR = Risk ratio, STI = Sexually transmitted infection, UMIC = Upper-middle-income country.

# **Supplementary Table S9.** Studies reporting proportions of HSV-2 virus isolation in clinically diagnosed genital ulcer disease and in clinically diagnosed genital herpes in Asia.

| **Author, year** | **Year(s) of data collection** | **Country** | **Study site** | **Original study design^a^** | **Sampling method** | **Population** | **HSV-2 biological assay** | **Sample size** | **Proportion of HSV-2 detection (%)** |
| --- | --- | --- | --- | --- | --- | --- | --- | --- | --- |
| **Patients with clinically diagnosed GUD** | | |  |  |  |  |  |  |  |
| Becker, 2010^127^ | 2004-04 | India | Outpatient clinic | CS | Conv | Males with GUD | PCR | 206 | 26.7 |
| Becker, 2010^127^ | 2004-04 | India | Outpatient clinic | CS | Conv | Females with GUD | PCR | 66 | 36.4 |
| Brijwal, 2019^156^ | 2017-18 | India | Outpatient clinic | CS | Conv | Patients with GUD | PCR | 43 | 27.9 |
| Chu, 2006^148^ | 2000-01 | Thailand | Community | CS | Conv | HIV-positive males | PCR | 11 | 100.0 |
| Chu, 2006^148^ | 2000-01 | Thailand | Community | CS | Conv | HIV-positive females | PCR | 15 | 100.0 |
| Chua, 1995^157^ | 1993-93 | Singapore | Outpatient clinic | CS | Conv | Patients with GUD | Culture | 531 | 48.4 |
| Hooi, 2002^158^ | 1990-99 | Malaysia | Hospital | CS | Conv | Patients with genital lesions in university clinic | IF | 99 | 25.3 |
| Hooi, 2002^158^ | 1990-99 | Malaysia | Hospital | CS | Conv | Patients with genital lesions in STI clinic | IF | 204 | 77.5 |
| Patwardhan, 2016^128^ | - | India | Outpatient clinic | CS | Conv | Patients with primary genital herpes | PCR | 21 | 71.4 |
| Patwardhan, 2016^128^ | - | India | Outpatient clinic | CS | Conv | Patients with recurrent genital herpes | PCR | 23 | 69.6 |
| Rajan, 1982^159^ | - | Singapore | Outpatient clinic | CS | Conv | Males with GUD | Culture | 22 | 4.6 |
| Sen, 2008^160^ | 2001-01 | Singapore | Outpatient clinic | CS | Conv | Patients with genital lesions | PCR | 103 | 48.5 |
| Thirumoorthy, 1986^161^ | 1984-84 | Singapore | Outpatient clinic | CS | Conv | Men with penile ulcers | IF | 80 | 11.3 |
| Zainah, 1991^162^ | 1989-90 | Malaysia | Outpatient clinic | CS | Conv | Patients with GUD | IF | 249 | 19.2 |
| **Patients with clinically diagnosed genital herpes** | | | | | | | | | |
| Becker, 2010^127^ | 2004-04 | India | Outpatient clinic | CS | Conv | Males with genital herpes | PCR | 18 | 33.3 |
| Becker, 2010^127^ | 2004-04 | India | Outpatient clinic | CS | Conv | Females with genital herpes | PCR | 45 | 68.9 |
| Bhattarakosol, 2005^163^ | 1998-04 | Thailand | Outpatient clinic | CS | Conv | Genital specimens from Thai patients | IF | 123 | 61.0 |
| Bhattarakosol, 2005^163^ | 1998-04 | Thailand | Outpatient clinic | CS | Conv | Genital specimens from foreigner patients | IF | 57 | 63.2 |
| Brijwal, 2019^156^ | 2017-18 | India | Outpatient clinic | CS | Conv | Patients with genital herpes | PCR | 16 | 75.0 |
| Cheong, 1990^164^ | 1986-87 | Singapore | Outpatient clinic | CS | Conv | Overall patients with first episode genital herpes | IF | 62 | 66.1 |
| Chiam, 2010^165^ | 1982-08 | Malaysia | Outpatient clinic | CS | Conv | Patients with suspected genital herpes | IF | 118 | 57.6 |
| Chio, 2015^166^ | 2014-14 | Singapore | Outpatient clinic | CS | Conv | Patients with genital herpes | PCR | 84 | 67.9 |
| Chua, 1995^157^ | 1993-93 | Singapore | Outpatient clinic | CS | Conv | Overall first episode genital herpes | Culture | 150 | 83.3 |
| Chua, 1995^157^ | 1993-93 | Singapore | Outpatient clinic | CS | Conv | Overall recurrent genital herpes | Culture | 135 | 97.8 |
| Doraisingham, 1987^167^ | 1984-86 | Singapore | Outpatient clinic | CS | Conv | Patients with genital lesions | IF | 215 | 78.6 |
| Doraisingham, 1987^167^ | 1984-86 | Singapore | Outpatient clinic | CS | Conv | Genital herpes isolates | IF | 46 | 65.2 |
| Goto, 1993^168^ | - | Japan | Outpatient clinic | CS | Conv | Lesions from females tested by PCR | Culture | 29 | 65.5 |
| Hashido, 1997b^80^ | 1972-85 | Japan | Outpatient clinic | CS | Conv | Women with acute genital herpes | IF | 30 | 36.7 |
| Hashido, 1997b^80^ | 1972-85 | Japan | Outpatient clinic | CS | Conv | Women with recurrent genital herpes | IF | 25 | 88.0 |
| Hashido, 1997b^80^ | 1972-85 | Japan | Outpatient clinic | CS | Conv | Women with provoked^b^ genital herpes | IF | 13 | 69.2 |
| Hooi, 2002^158^ | 1990-99 | Malaysia | Hospital | CS | Conv | Patients with HSV seen in a university hospital | IF | 55 | 47.3 |
| Hooi, 2002^158^ | 1990-99 | Malaysia | Hospital | CS | Conv | Patients with HSV in a STI clinic | IF | 165 | 95.8 |
| Ishiguro, 1982^169^ | 1975-78 | Japan | Outpatient clinic | CS | Conv | Women with genital herpes | NA | 13 | 46.2 |
| Jacob, 1989^170^ | 1983-86 | India | Outpatient clinic | CS | Conv | Patients with 1st episode genital herpes | Mab | 10 | 90.0 |
| Jacob, 1989^170^ | 1983-86 | India | Outpatient clinic | CS | Conv | Patients with recurring genital herpes | Mab | 52 | 100.0 |
| Kaneko, 2005^171^ | - | Japan | Outpatient clinic | CS | Conv | Females with genital herpes | PCR | 30 | 43.3 |
| Kao, 1991^172^ | 1981-90 | Taiwan | Hospital | CS | Conv | Genital HSV isolates | IF | 149 | 94.0 |
| Kawana, 1982^173^ | - | Japan | Outpatient clinic | CS | Conv | Women with genital herpes | NA | 88 | 59.1 |
| Mathew, 2018^174^ | - | India | Outpatient clinic | CS | Conv | Patients with genital herpes | PCR | 33 | 42.4 |
| Sen, 2008^160^ | 2001-01 | Singapore | Outpatient clinic | CS | Conv | Patients with genital herpes | PCR | 63 | 79.4 |
| Theng, 2004^175^ | 2001-01 | Singapore | Outpatient clinic | Cohort | Conv | Patients with HSV | IF | 241 | 88.4 |

^a^ The reported study design is the original study design (cross sectional or cohort). The included seroprevalence measures are those for the baseline measures at the beginning of the study.

^b^ Provoked genital herpes was defined in the article as herpetic lesions induced during or following an immunosuppressive event.

Abbreviations: Conv = Convenience, CS = Cross sectional, FSWs = Female sex workers, GUD = Genital ulcer disease, HSV-2 = Herpes simplex virus type 2, IF = Immunofluorescence, Mab = Monoclonal antibody, Nab = Neutralization assay, PCR = Polymerase chain reaction, STI = Sexually transmitted infection.

# **Supplementary Figure S2.** Forest plots presenting outcomes of the pooled mean proportions of HSV-2 virus isolation in clinically diagnosed genital ulcer disease and in clinically diagnosed genital herpes in Asia.

##
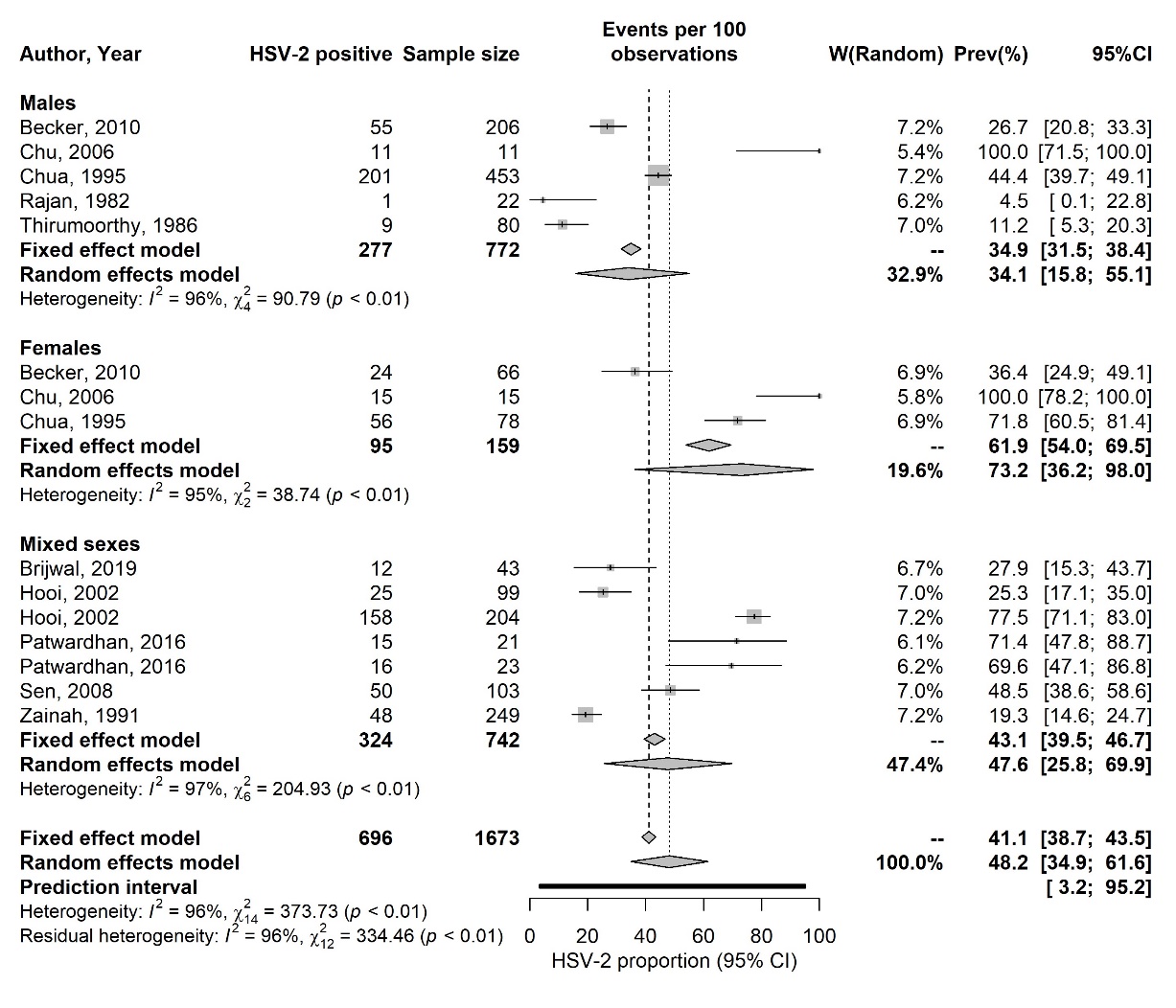
Patients with genital ulcer disease

##
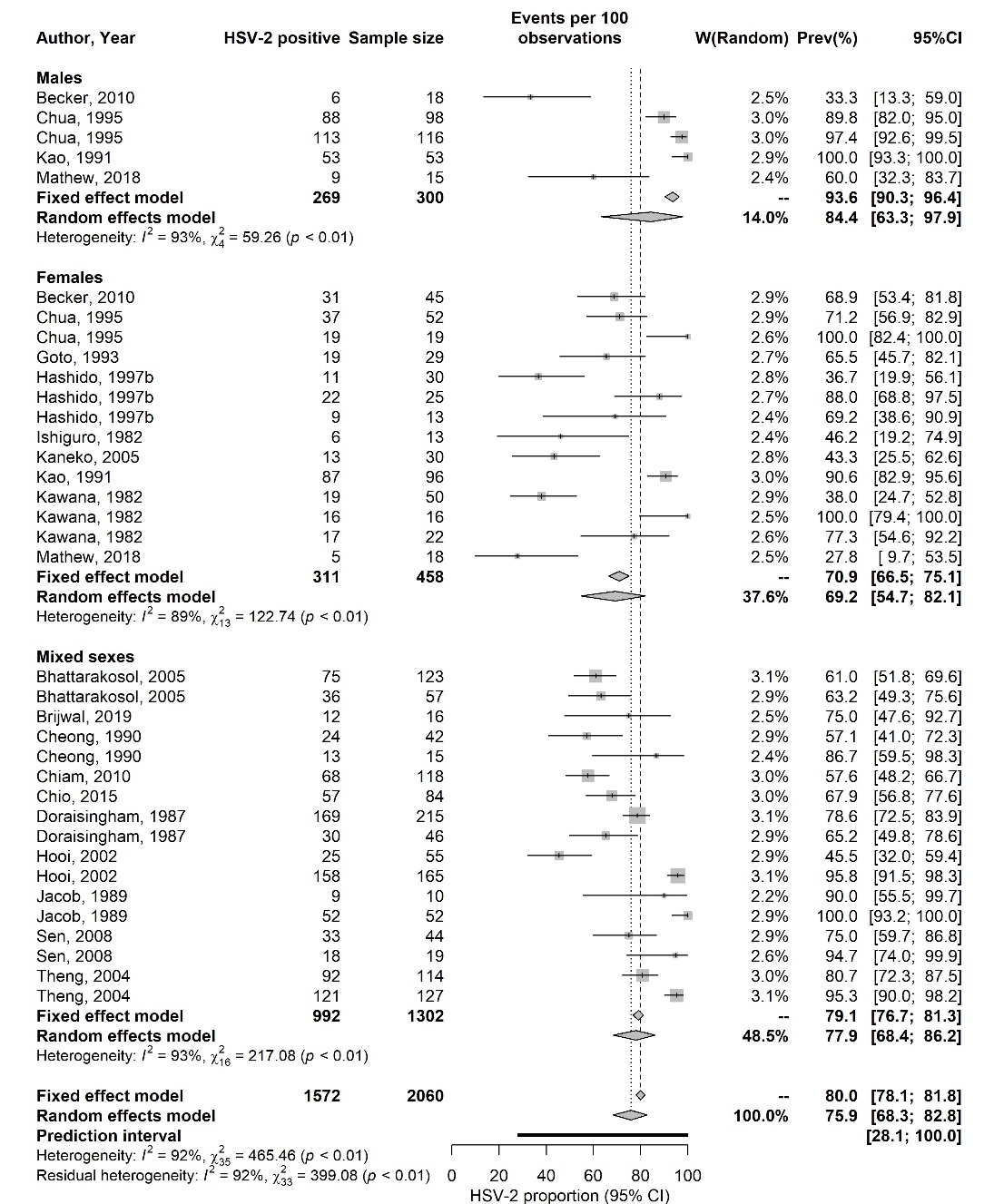
Patients with genital herpes

# **Supplementary Table S10.** Summary of precision assessment and risk of bias (ROB) assessment for studies reporting HSV-2 seroprevalence in Asia.

| **Quality assessment** | **HSV-2 seroprevalence measures** | |
| --- | --- | --- |
|  | **Number of studies** | **%** |
| **Precision of seroprevalence measures^a^** | | |
| Low precision | 63 | 23.2 |
| High precision | 209 | 76.8 |
| **Risk of bias quality domain^b^** | | |
| **Sampling method** | | |
| Low risk of bias | 74 | 27.2 |
| High risk of bias | 198 | 72.3 |
| **Response rate** | | |
| Low risk of bias | 50 | 18.4 |
| High risk of bias | 49 | 18.0 |
| Unclear risk of bias | 173 | 63.6 |
| **Summary of the risk of bias assessment** | | |
| **Low risk of bias** |  |  |
| In at least one quality domain | 75 | 27.5 |
| In both quality domains | 23 | 8.4 |
| **High risk of bias** |  |  |
| In at least one quality domain | 76 | 27.9 |
| In both quality domains | 24 | 8.8 |
| **Seroprevalence studies where risk of bias assessment was possible** | **272** | **100** |

^a^ Precision was assessed based on the overall sample size (not each stratum subsample size) of the study as reported in the record/publication.

^b^ Risk of bias was assessed based on the overall sample size (not each stratum subsample size) of the study as reported in the record/publication.

Abbreviations: HSV-2 = Herpes simplex virus type 2.

# **References**

1 Moher, D., Liberati, A., Tetzlaff, J., Altman, D. G. & Group, P. Preferred reporting items for systematic reviews and meta-analyses: the PRISMA statement. *PLoS Med* **6**, e1000097, doi:10.1371/journal.pmed.1000097 (2009).

2 *World Health Organization. WHO regional offices*, <<http://www.who.int/about/regions/en/>> (

3 World Bank. *World Bank Country and Lending Groups (Available at:* [*https://datahelpdesk.worldbank.org/knowledgebase/articles/906519-world-bank-country-and-lending-groups*](https://datahelpdesk.worldbank.org/knowledgebase/articles/906519-world-bank-country-and-lending-groups)*. Accessed in June 2017)*, 2017).

4 Bogaerts, J. *et al.* Sexually transmitted infections among married women in Dhaka, Bangladesh: unexpected high prevalence of herpes simplex type 2 infection. *Sex Transm Infect* **77**, 114-119, doi:DOI 10.1136/sti.77.2.114 (2001).

5 Hochberg, C. H. *et al.* Population and dyadic-based seroincidence of herpes simplex virus-2 and syphilis in southern India. *Sex Transm Infect* **91**, 375-382, doi:<http://0-dx.doi.org.elibrary.qatar-weill.cornell.edu/10.1136/sextrans-2014-051708> (2015).

6 Madhivanan, P. *et al.* Incidence of herpes simplex virus type 2 in young reproductive age women in Mysore, India. *Indian J Pathol Microbiol.* **54**, 96-99. doi: 10.4103/0377-4929.77336. (2011).

7 Sutcliffe, C. G. *et al.* Incidence of HIV and sexually transmitted infections and risk factors for acquisition among young methamphetamine users in northern Thailand. *Sex Transm Dis.* **36**, 284-289. doi: 210.1097/OLQ.1090b1013e318191ba318117. (2009).

8 Li, D. *et al.* HIV incidence among men who have sex with men in Beijing: a prospective cohort study. *BMJ Open.* **2(6).** e001829. doi: 001810.001136/bmjopen-002012-001829. Print 002012. (2012).

9 Thienkrua, W. *et al.* Incidence of and temporal relationships between HIV, herpes simplex II virus, and syphilis among men who have sex with men in Bangkok, Thailand: an observational cohort. *BMC Infect Dis.* **16:340.**, 10.1186/s12879-12016-11667-z. (2016).

10 Wang, H. *et al.* Herpes simplex virus type 2 incidence and associated risk factors among female sex workers in a high HIV-prevalence area of China. *Int J STD AIDS.* **24**, 441-446. doi: 410.1177/0956462412472800. Epub 0956462412472013 Jun 0956462412472824. (2013).

11 Bollen, L. J. *et al.* Maternal herpes simplex virus type 2 coinfection increases the risk of perinatal HIV transmission: possibility to further decrease transmission? *AIDS.* **22**, 1169-1176. doi: 1110.1097/QAD.1160b1013e3282fec1142a. (2008).

12 Ding, Y. *et al.* Risk factors for incident HSV-2 infections among a prospective cohort of HIV-1-discordant couples in China. *Sex Transm Infect.* **92**, 76-82. doi: 10.1136/sextrans-2014-051975. Epub 052015 Jul 051972. (2016).

13 Kumarasamy, N. *et al.* Prevalence and incidence of sexually transmitted infections among South Indians at increased risk of HIV infection. *AIDS Patient Care STDS.* **22**, 677-682. doi: 610.1089/apc.2007.0166. (2008).

14 Reynolds, S. J. *et al.* Recent Herpes Simplex Virus Type 2 Infection and the Risk of Human Immunodeficiency Virus Type 1 Acquisition in India. *J Infect Dis.* **187**, 1513-1521. Epub 2003 Apr 1523. (2003).

15 Reynolds, S. J. *et al.* Male circumcision and risk of HIV-1 and other sexually transmitted infections in India. *Lancet* **363**, 1039–1040, doi:10.1016/S0140-6736(04)15840-6 (2004).

16 Vallely, A. *et al.* High prevalence and incidence of HIV, sexually transmissible infections and penile foreskin cutting among sexual health clinic attendees in Papua New Guinea. *Sex Health.* **11**, 58-66. doi: 10.1071/SH13197. (2014).

17 Ashley-Morrow, R., Nollkamper, J., Robinson, N. J., Bishop, N. & Smith, J. Performance of focus ELISA tests for herpes simplex virus type 1 (HSV-1) and HSV-2 antibodies among women in ten diverse geographical locations. *Clin Microbiol Infec* **10**, 530-536, doi:10.1111/j.1469-0691.2004.00836.x (2004).

18 Chen, X. S. *et al.* Herpes simplex virus 2 infection in women attending an antenatal clinic in Fuzhou, China. *Sex Transm Infect* **83**, 369-370, doi:10.1136/sti.2007.025452 (2007).

19 Chen, L. *et al.* Seasonal influence on TORCH infection and analysis of multi-positive samples with indirect immunofluorescence assay. *Journal of clinical laboratory analysis* **33**, e22828, doi:10.1002/jcla.22828 (2019).

20 Doi, Y. *et al.* Seroprevalence of herpes simplex virus 1 and 2 in a population-based cohort in Japan. *J Epidemiol* **19**, 56-62, doi:10.2188/jea.je20080061 (2009).

21 Guan, X. R. *et al.* Respiratory syncytial virus infection and risk of acute myocardial infarction. *American Journal of the Medical Sciences* **340**, 356-359, doi:<http://dx.doi.org/10.1097/MAJ.0b013e3181eecf29> (2010).

22 Hashido, M. *et al.* An epidemiologic study of herpes simplex virus type 1 and 2 infection in Japan based on type-specific serological assays. *Minerva Ginecol.* **50**, 105-107. (1998a).

23 Hashido, M., Kawana, T., Matsunaga, Y. & Inouye, S. Changes in prevalence of herpes simplex virus type 1 and 2 antibodies from 1973 to 1993 in the rural districts of Japan. *Indian J Exp Biol.* **36**, 967-972. (1998b).

24 He, N. *et al.* Herpes simplex virus-2 infection in male rural migrants in Shanghai, China. *Int J STD AIDS.* **20**, 112-114. doi: 110.1258/ijsa.2008.008217. (2009).

25 Huai, P. *et al.* Seroprevalence and associated factors of HSV-2 infection among general population in Shandong Province, China. *BMC infectious diseases* **19**, 382, doi:10.1186/s12879-019-3995-2 (2019).

26 Itoh, N. *et al.* High prevalence of herpes simplex virus type 2 in acute retinal necrosis syndrome associated with herpes simplex virus in Japan. *Phytomedicine.* **6**, 411-419. (2000).

27 Kim, O. *et al.* Seroprevalence of sexually transmitted viruses in Korean populations including HIV-seropositive individuals. *International Journal of STD and AIDS* **14**, 46-49, doi:<http://0-dx.doi.org.elibrary.qatar-weill.cornell.edu/10.1258/095646203321043264> (2003).

28 Lee, A. & Lee, K. Type-specific herpes simplex virus-1 and herpes simplex virus-2 seroprevalence in Korea. *International Journal of Antimicrobial Agents* **45**, S138 (2015).

29 Li, J. M., Chen, Y. R., Li, X. T. & Xu, W. C. Screening of Herpes simplex virus 2 infection among pregnant women in southern China. *J Dermatol.* **38**, 120-124. doi: 110.1111/j.1346-8138.2010.00966.x. Epub 02010 Sep 00962. (2011).

30 Li, Z., Yan, R., Yan, C., Liu, P. & Feng, Z. Evaluation of an Automated Chemiluminescent Immunoassay in Typing Detection of IgG Antibodies Against Herpes Simplex Virus. *Journal of clinical laboratory analysis*, doi:<http://0-dx.doi.org.elibrary.qatar-weill.cornell.edu/10.1002/jcla.21905> (2016a).

31 Lin, H. *et al.* Herpes simplex virus infections among rural residents in eastern China. *BMC Infect Dis.* **11:69.**, 10.1186/1471-2334-1111-1169. (2011).

32 Lo, J. Y., Lim, W. W., Ho, D. W., Field, P. R. & Cunningham, A. L. Difference in seroprevalence of herpes simplex virus type 2 infection among antenatal women in Hong Kong and southern China. *Biochem Biophys Res Commun.* **263**, 352-356. (1999).

33 Nakagawa, M., Harada, S., Ban, F., Yanagi, K. & Osame, M. High prevalence of herpes virus type 2 infection in Okinawa. *Vaccine.* **9**, 147-153. (1991).

34 Peng, H. Q., Liu, S. L., Mann, V., Rohan, T. & Rawls, W. Human papillomavirus types 16 and 33, herpes simplex virus type 2 and other risk factors for cervical cancer in Sichuan Province, China. *Arzneimittelforschung.* **41**, 549-552. (1991).

35 Shen, J. H. *et al.* Seroprevalence of Herpes Simplex Virus Type 1 and 2 in Taiwan and Risk Factor Analysis, 2007. *PLoS One.* **10**, e0134178. doi: 0134110.0131371/journal.pone.0134178. eCollection 0132015. (2015).

36 Shin, H. R. *et al.* Prevalence of human papillomavirus infection in women in Busan, South Korea. *Int J Cancer* **103**, 413-421, doi:10.1002/ijc.10825 (2003).

37 Shin, H. *et al.* Herpes simplex virus type 2 seroprevalence in Korea: Rapid increase of HSV-2 seroprevalence in the 30s in the southern part. *Journal of Korean Medical Science* **22**, 957-962, doi:<http://0-dx.doi.org.elibrary.qatar-weill.cornell.edu/10.3346/jkms.2007.22.6.957> (2007).

38 Wang, L. C., Yan, F., Ruan, J. X., Xiao, Y. & Yu, Y. TORCH screening used appropriately in China?─three years results from a teaching hospital in northwest China. *BMC pregnancy and childbirth* **19**, 484, doi:10.1186/s12884-019-2642-7 (2019).

39 Yongjun, T. *et al.* The prevalence of sexually transmitted and other lower reproductive tract infections among rural women in Sichuan Province, China. *Southeast Asian J Trop Med Public Health.* **40**, 1038-1047. (2009).

40 Zhang, J. F. & Zhang, W. Y. Relationship of cytomegalovirus, Chlamydia pneumoniae and herpes simplex virus type 2 infections with preeclampsia. *National Medical Journal of China* **92**, 1413-1415, doi:<http://0-dx.doi.org.elibrary.qatar-weill.cornell.edu/10.3760/cma.j.issn.0376-2491.2012.20.013> (2012).

41 Zhang, T. *et al.* Kaposis sarcoma associated herpesvirus infection among female sex workers and general population women in Shanghai, China: a cross-sectional study. *BMC infectious diseases* **14**, 58 (2014).

42 Chen, X. S. *et al.* Prevalence of sexually transmitted infections among long-distance truck drivers in Tongling, China. *Int J Std Aids* **17**, 304-308, doi:Doi 10.1258/095646206776790141 (2006).

43 Reilly, K. H. *et al.* HIV and associated risk factors among male clients of female sex workers in a Chinese border region. *Sex Transm Dis.* **39**, 750-755. (2012).

44 Wei, S. B. *et al.* A study of commercial sex and HIV/STI-related risk factors among hospitality girls in entertainment establishments in Wuhan, China. *Sex Health* **1**, 141-144. (2004).

45 Wu, Z. *et al.* Sexually transmitted diseases and risk behaviors among market vendors in China. *Sexually Transmitted Diseases* **34**, 1030-1034, doi:<http://0-dx.doi.org.elibrary.qatar-weill.cornell.edu/10.1097/OLQ.0b013e318141fe89> (2007).

46 Xu, J. J. *et al.* HIV and STIs in clients and female sex workers in mining regions of Gejiu City, China. *Sex Transm Dis.* **35**, 558-565. doi: 510.1097/OLQ.1090b1013e318165926b. (2008).

47 Zhu, J. *et al.* HIV prevalence and correlated factors among male clients of female sex workers in a border region of China. *PloS one* **14**, e0225072, doi:10.1371/journal.pone.0225072 (2019).

48 Chen, X. S. *et al.* Sexually transmitted infections among female sex workers in Yunnan, China. *Aids Patient Care St* **19**, 853-860, doi:DOI 10.1089/apc.2005.19.853 (2005).

49 Chen, S. C. *et al.* Seropositivity and Risk Factors for Herpes Simplex Virus Type 2 Infection among Female Sex Workers in Guangxi, China. *PloS one* **8**, e69697. doi: 69610.61371/journal.pone.0069697. Print 0062013., doi:ARTN e6969710.1371/journal.pone.0069697 (2013).

50 Chen, X. *et al.* Club Drugs and HIV/STD Infection: An Exploratory Analysis among Men Who Have Sex with Men in Changsha, China. *PloS one* **10**, e0126320. doi: 0126310.0121371/journal.pone.0126320. eCollection 0122015., doi:ARTN e012632010.1371/journal.pone.0126320 (2015).

51 Ding, Y., Zhou, Y., Liu, C., Liu, X. & He, N. Sex with older partners, condomless anal sex and unrecognized HIV infection among Chinese men who have sex with men. *AIDS Care* **5**, 1-7 (2017).

52 Feng, Y. *et al.* HIV/STD prevalence among men who have sex with men in Chengdu, China and associated risk factors for HIV infection. *J Acquir Immune Defic Syndr.* **53**, S74-80. doi: 10.1097/QAI.1090b1013e3181c1097dd1016. (2010).

53 Gao, Y. *et al.* Prevalence and predictors of HIV, syphilis and herpes simplex type 2 virus (HSV-2) infections among the men who have sex with men (MSM) in Beijing. *Chinese Journal of Public Health* **28**, 451-453 (2012).

54 Han, L. *et al.* Differences in risk behaviours and HIV/STI prevalence between low-fee and medium-fee female sex workers in three provinces in China. *Sex Transm Infect.* **92**, 309-315. doi: 310.1136/sextrans-2015-052173. Epub 052015 Oct 052116. (2016).

55 Hu, Q. H. *et al.* Prevalence and Determinants of Herpes Simplex Virus Type 2 (HSV-2)/Syphilis Co-Infection and HSV-2 Mono-Infection among Human Immunodeficiency Virus Positive Men Who Have Sex with Men: a Cross-Sectional Study in Northeast China. *Jpn J Infect Dis.* **70**, 284-289. doi: 210.7883/yoken.JJID.2016.7177. Epub 2016 Oct 7831. (2017).

56 Jiang, J. *et al.* High prevalence of sexually transmitted diseases among men who have sex with men in Jiangsu Province, China. *Sex Transm Dis.* **33**, 118-123. (2006).

57 Li, D. *et al.* Incidence of Co-Infections of HIV, Herpes Simplex Virus Type 2 and Syphilis in a Large Cohort of Men Who Have Sex with Men in Beijing, China. *PLoS One.* **11**, e0147422. doi: 0147410.0141371/journal.pone.0147422. eCollection 0142016. (2016).

58 Liu, S. *et al.* A survey of condom use behaviors and HIV/STI prevalence among venue-based money boys in Shenzhen, China. *AIDS Behav.* **16**, 835-846. doi: 810.1007/s10461-10011-19978-y. (2012a).

59 Liu, Z. *et al.* High seroprevalence of human herpesvirus 8 and herpes simplex virus 2 infections in men who have sex with men in Shanghai, China. *J Med Virol.* **89**, 887-894. doi: 810.1002/jmv.24718. Epub 22016 Nov 24719. (2017a).

60 Liu, Y. *et al.* [Co-infections of HIV, syphilis and HSV-2 among men who have sex with men at the voluntary HIV counseling and testing clinics in Shanghai]. *Zhonghua liu xing bing xue za zhi = Zhonghua liuxingbingxue zazhi* **38**, 1363-1366, doi:10.3760/cma.j.issn.0254-6450.2017.10.013 (2017b).

61 Luo, L., Li, X. & Zhang, L. L. Neisseria gonorrhoeae prevalence, incidence and associated risk factors among female sex workers in a high HIV-prevalence area of China. *Int J Infect Dis* **38**, 115-120, doi:10.1016/j.ijid.2015.07.025 (2015).

62 Mao, X. *et al.* HIV incidence is rapidly increasing with age among young men who have sex with men in China: a multicentre cross-sectional survey. *HIV Medicine* **19**, 513-522, doi:<http://0-dx.doi.org.elibrary.qatar-weill.cornell.edu/10.1111/hiv.12623> (2018).

63 Ngo, T. D. *et al.* Herpes simplex virus type 2 infection among commercial sex workers in Kunming, Yunnan Province, China. *Int J STD AIDS.* **19**, 694-697. doi: 610.1258/ijsa.2008.008072. (2008a).

64 Wang, J. J. *et al.* Estimation of population-size changes and HIV prevalence among female sex workers from 2006 to 2009 in Kaiyuan, Yunnan, China. *Biomed Environ Sci.* **25**, 489-494. doi: 410.3967/0895-3988.2012.3904.3016. (2012a).

65 Wang, J. J. *et al.* Herpes simplex virus type 2 risks in female sex workers in the China-Vietnam border county of Hekou. *Biomed Environ Sci.* **25**, 706-710. doi: 710.3967/0895-3988.2012.3906.3013. (2012b).

66 Xu, J. *et al.* Factors associated with HIV testing history and HIV-test result follow-up among female sex workers in two cities in Yunnan, China. *Sexually Transmitted Diseases* **38**, 89-95, doi:<http://dx.doi.org/10.1097/OLQ.0b013e3181f0bc5e> (2011).

67 Xu, J. J. *et al.* High HIV incidence epidemic among men who have sex with men in china: results from a multi-site cross-sectional study. *Infect Dis Poverty.* **5**, 82. doi: 10.1186/s40249-40016-40178-x. (2016).

68 Yan, H. *et al.* Increased HIV Testing among Men Who Have Sex with Men from 2008 to 2012, Nanjing, China. *PLoS One.* **11**, e0154466. doi: 0154410.0151371/journal.pone.0154466. eCollection 0152016. (2016).

69 Yang, Y. *et al.* Herpes simplex virus type 2 infection among female sex workers in Shanghai, China. *AIDS Care.* **23**, 37-44. doi: 10.1080/09540121.09542011.09555740. (2011).

70 Yao, Y. *et al.* Associations between drug use and risk behaviours for HIV and sexually transmitted infections among female sex workers in Yunnan, China. *Int J STD AIDS.* **23**, 698-703. doi: 610.1258/ijsa.2012.011346. (2012).

71 Yin, Y. P. *et al.* Prevalence and risk factors of HSV-2 infection and HSV-2/HIV coinfection in men who have sex with men in China: a multisite cross-sectional study. *Sex Transm Dis.* **39**, 354-358. doi: 310.1097/OLQ.1090b1013e318244aef318240. (2012).

72 Yun, H. *et al.* Prevalence of human papillomavirus and herpes simplex virus type 2 infection in Korean commercial sex workers. *J Microbiol Biotechnol.* **18**, 350-354. (2008).

73 Zhang, T. *et al.* Prevalence and correlates of Kaposi's sarcoma-associated herpesvirus infection in a sample of men who have sex with men in Eastern China. *Epidemiology and infection* **141**, 1823-1830, doi:10.1017/s0950268812002361 (2013).

74 Zhu, W. M. *et al.* [Human immunodeficiency virus/sexually transmitted infection, risk behavior and sexual networks among men who have sex with men in Taizhou city, Zhejiang province]. *Zhonghua Liu Xing Bing Xue Za Zhi.* **29**, 994-998. (2008).

75 Zhu, J. *et al.* [Consecutive cross-sectional survey of prevalence of HIV infection/STD and related factors in Vietnamese female sex workers at a China-Vietnam border area, 2014-2015]. *Zhonghua Liu Xing Bing Xue Za Zhi.* **38**, 638-642. doi: 610.3760/cma.j.issn.0254-6450.2017.3705.3016. (2017a).

76 Zhu, J. *et al.* HIV prevalence and correlated factors of female sex workers and male clients in a border region of Yunnan Province, China. *Int J Std Aids* **1**, 0956462417730258 (2017b).

77 Chen, L. *et al.* [Prevalence and correlates of herpes simplex virus infections among AIDS patients in a county of Shanxi province, China]. *Zhonghua yu fang yi xue za zhi [Chinese journal of preventive medicine]* **44**, 526-530 (2010).

78 Fu, Z. H. *et al.* [Study on herpes simplex virus 2 infection and risky sexual behavior among HIV-infected patients in a county of Yunnan province]. *Zhonghua Liu Xing Bing Xue Za Zhi.* **30**, 1139-1142. (2009).

79 He, N. *et al.* Multiple viral coinfections among HIV/AIDS patients in China. *Biosci Trends* **5**, 1-9, doi:10.5582/bst.2011.v5.1.1 (2011).

80 Hashido, M., Lee, F. K., Nahmias, A. J. & Kawana, T. Prevalence of herpes simplex virus type 1-and 2-specific antibodies among the acute, recurrent, and provoked types of female genital herpes. *Microbiology and immunology* **41**, 823-827 (1997).

81 Yin, Y. P. *et al.* Performance of the Focus ELISA test for detection of herpes simplex virus type-2-specific antibodies in Chinese STD patients. *Clin Microbiol Infect.* **11**, 423-424. (2005).

82 Wang, J. S. *et al.* Hospital-based HIV/HSV-2 seroprevalence among male patients with anal disease in Korea: cross sectional study. *BMC Infect Dis.* **14:34.**, 10.1186/1471-2334-1114-1134. (2014).

83 Adamson, P. C. *et al.* Prevalence & correlates of primary infertility among young women in Mysore, India. *Indian J Med Res* **134**, 440-446 (2011).

84 Banandur, P. *et al.* Heterogeneity of the HIV epidemic in the general population of Karnataka state, south India. *BMC Public Health* **11 Suppl 6**, S13, doi:10.1186/1471-2458-11-S6-S13 (2011).

85 Becker, M. L. *et al.* Prevalence and determinants of HIV infection in South India: a heterogeneous, rural epidemic. *AIDS* **21**, 739-747, doi:10.1097/QAD.0b013e328012b885 (2007).

86 Biswas, D. *et al.* Seroprevalence and risk factors of herpes simplex virus type-2 infection among pregnant women in Northeast India. *BMC infectious diseases* **11**, 10.1186/1471-2334-1111-1325., doi:Artn 32510.1186/1471-2334-11-325 (2011).

87 Bochner, A. F. *et al.* The Epidemiology of Herpes Simplex Virus Type-2 Infection among Pregnant Women in Rural Mysore Taluk, India. *Journal of sexually transmitted diseases* **2013**, 750415, doi:10.1155/2013/750415 (2013).

88 Chawla, R., Bhalla, P., Bhalla, K., Singh, M. M. & Garg, S. Community-based study on seroprevalence of herpes simplex virus type 2 infection in New Delhi. *Indian J Med Microbiol* **26**, 34-39, doi:10.4103/0255-0857.38855 (2008).

89 Coudray, M. *et al.* Age disparity and sociodemographic correlates of herpes simplex virus type 2 (HSV-2) seropositivity in South India. *Sex Transm Infect* **95**, A115, doi:<http://dx.doi.org/10.1136/sextrans-2019-sti.290> (2019).

90 Cowan, F. M. *et al.* Seroepidemiological study of herpes simplex virus types 1 and 2 in Brazil, Estonia, India, Morocco, and Sri Lanka. *Sex Transm Infect* **79**, 286-290, doi:<http://0-dx.doi.org.elibrary.qatar-weill.cornell.edu/10.1136/sti.79.4.286> (2003).

91 Das, S., Yemul, V. & Deshmukh, R. Incidence and association of HIV and other STDs in 200 persons belonging to a high risk group in central Mumbai. *Venereology : official publication of the National Venereology Council of Australia* **11**, 19-23 (1998).

92 Munawwar, A., Gupta, S., Sharma, S. K. & Singh, S. Seroprevalence of HSV-1 and 2 in HIV-infected males with and without GUD: Study from a tertiary care setting of India. *Journal of laboratory physicians* **10**, 326-331, doi:10.4103/jlp.Jlp_7_18 (2018).

93 Munro, H. L. *et al.* Prevalence and determinants of HIV and sexually transmitted infections in a general population-based sample in Mysore district, Karnataka state, southern India. *Aids* **22**, S117-S125 (2008).

94 Nag, S. *et al.* Seroprevalence of Herpes Simplex Virus Infection in HIV Coinfected Individuals in Eastern India with Risk Factor Analysis. *Advances in virology* **2015**, 537939, doi:10.1155/2015/537939 (2015).

95 Nasrallah, G., Dargham, S., Harfouche, M. & Abu-Raddad, L. Seroprevalence of Herpes simplex virus types 1 and 2 in Indian and Filipino migrant populations in Qatar: a cross-sectional survey. *East Mediterr Health J* **26**, 609-615, doi:10.26719/2020.26.5.609 (2020).

96 Panchanadeswaran, S. *et al.* Gender differences in the prevalence of sexually transmitted infections and genital symptoms in an urban setting in southern India. *Sex Transm Infect* **82**, 491-495, doi:<http://0-dx.doi.org.elibrary.qatar-weill.cornell.edu/10.1136/sti.2006.020768> (2006).

97 Raj, R., Sreenivas, V., Mehta, M. & Gupta, S. Health-related quality of life in Indian patients with three viral sexually transmitted infections: herpes simplex virus-2, genital human papilloma virus and HIV. *Sex Transm Infect.* **87**, 216-220. doi: 210.1136/sti.2010.043356. Epub 042011 Feb 043352. (2011).

98 Rajaram, S. P. *et al.* Is HIV prevalence declining in southern India? Evidence from two rounds of general population surveys in Bagalkot district, Karnataka. *Sex Transm Infect* **87**, A105, doi:<http://0-dx.doi.org.elibrary.qatar-weill.cornell.edu/10.1136/sextrans-2011-050108.14> (2011).

99 Rathore, S., Jamwal, A. & Gupta, V. Herpes simplex virus type 2: Seroprevalence in antenatal women. *Indian journal of sexually transmitted diseases* **31**, 11 (2010).

100 Schensul, S. L. *et al.* Sexually transmitted infections in men in Mumbai slum communities: the relationship of prevalence to risk behavior. *Sex Transm Dis.* **34**, 444-450. (2007).

101 Schneider, J. A. *et al.* Initial commitment to pre-exposure prophylaxis and circumcision for HIV prevention amongst Indian truck drivers. *PLoS One.* **5**, e11922. doi: 11910.11371/journal.pone.0011922. (2010a).

102 Sgaier, S. K. *et al.* Prevalence and correlates of Herpes Simplex Virus-2 and syphilis infections in the general population in India. *Sex Transm Infect.* **87**, 94-100. doi: 110.1136/sti.2010.043687. Epub 042010 Nov 043688. (2011).

103 Celentano, D. D. *et al.* Prevalence of Sexually Transmitted Diseases and Risk Behaviors from the NIMH Collaborative HIV/STD Prevention Trial. *Int J Sex Health* **22**, 272-284, doi:10.1080/19317611.2010.494092 (2010).

104 Gibney, L. *et al.* Prevalence of infectious diseases in Bangladeshi women living adjacent to a truck stand: HIV/STD/hepatitis/genital tract infections. *Sex Transm Infect* **77**, 344-350, doi:<http://0-dx.doi.org.elibrary.qatar-weill.cornell.edu/10.1136/sti.77.5.344> (2001).

105 Gibney, L. *et al.* STD in Bangladesh's trucking industry: prevalence and risk factors. *Sex Transm Infect* **78**, 31-36 (2002).

106 Go, V. F. *et al.* HIV rates and risk behaviors are low in the general population of men in Southern India but high in alcohol venues: results from 2 probability surveys. *J Acquir Immune Defic Syndr.* **46**, 491-497. (2007).

107 Haseen, F. *et al.* Sexually transmitted infections and sexual behaviour among youth clients of hotel-based female sex workers in Dhaka, Bangladesh. *Int J STD AIDS.* **23**, 553-559. doi: 510.1258/ijsa.2012.011373. (2012).

108 National Summary Report - India. Integrated Behavioural and Biological Assessment (IBBA), Round 2 (2009-2010). Report No. ISBN 1-933702-81-8, (2011).

109 Panda, S. *et al.* Sexually transmitted infections and sexual practices in injection drug users and their regular sex partners in Chennai, India. *Sex Transm Dis.* **34**, 250-253. (2007).

110 Panda, S. *et al.* Alarming epidemics of human immunodeficiency virus and hepatitis C virus among injection drug users in the northwestern bordering state of Punjab, India: prevalence and correlates. *Int J STD AIDS.* **25**, 596-606. doi: 510.1177/0956462413515659. Epub 0956462413512013 Dec 0956462413515618. (2014).

111 Shaw, S. *et al.* Prevalence of hiv and sexually transmitted infections among clients of female sex workers in Karnataka, South India. *Sex Transm Infect* **87**, A122-A123, doi:<http://0-dx.doi.org.elibrary.qatar-weill.cornell.edu/10.1136/sextrans-2011-050108.58> (2011).

112 Uma, S. *et al.* Bacterial vaginosis in women of low socioeconomic status living in slum areas in Chennai, India. *Sex Health* **3**, 297-298, doi:10.1071/sh06036 (2006).

113 Barua, P. *et al.* Sexual activity as risk factor for hepatitis C virus (HCV) transmission among the female sex workers in Nagaland. *Indian J Med Res* **136**, 30-35 (2012).

114 Gutierrez, J. P., McPherson, S., Fakoya, A., Matheou, A. & Bertozzi, S. M. Community-based prevention leads to an increase in condom use and a reduction in sexually transmitted infections (STIs) among men who have sex with men (MSM) and female sex workers (FSW): the Frontiers Prevention Project (FPP) evaluation results. *BMC Public Health.* **10:497.**, 10.1186/1471-2458-1110-1497. (2010).

115 Mishra, S. *et al.* Sex work, syphilis, and seeking treatment: an opportunity for intervention in HIV prevention programming in Karnataka, South India. *Sex Transm Dis* **36**, 157-164, doi:10.1097/OLQ.0b013e31818d64e6 (2009).

116 Nessa, K. *et al.* Epidemiology and etiology of sexually transmitted infection among hotel-based sex workers in Dhaka, Bangladesh. *Journal of clinical microbiology* **42**, 618-621 (2004).

117 Qutub, M. & Akhter, J. Epidemiology of genital herpes (HSV-2) among brothel based female sex workers in Bangladesh. *European Journal of Epidemiology* **18**, 903-905, doi:<http://0-dx.doi.org.elibrary.qatar-weill.cornell.edu/10.1023/A:1025607913408> (2003).

118 Rahman, M. *et al.* Etiology of sexually transmitted infections among street-based female sex workers in Dhaka, Bangladesh. *Am J Ophthalmol.* **129**, 404-405. (2000).

119 Reza-Paul, S. *et al.* Declines in risk behaviour and sexually transmitted infection prevalence following a community-led HIV preventive intervention among female sex workers in Mysore, India. *Aids* **22**, S91-S100 (2008).

120 Sarna, A. *et al.* Sexually transmitted infections and reproductive health morbidity in a cohort of female sex workers screened for a microbicide feasibility study in Nellore, India. *Glob J Health Sci* **5**, 139-149, doi:10.5539/gjhs.v5n3p139 (2013).

121 Setia, M. S. *et al.* Men who have sex with men and transgenders in Mumbai, India: an emerging risk group for STIs and HIV. *Indian J Dermatol Venereol Leprol.* **72**, 425-431. (2006).

122 Shahmanesh, M. *et al.* The burden and determinants of HIV and sexually transmitted infections in a population-based sample of female sex workers in Goa, India. *Sex Transm Infect.* **85**, 50-59. doi: 10.1136/sti.2008.030767. Epub 032008 Aug 030766. (2009).

123 Solomon, S. S. *et al.* The emerging HIV epidemic among men who have sex with men in Tamil Nadu, India: geographic diffusion and bisexual concurrency. *AIDS Behav.* **14**, 1001-1010. doi: 1010.1007/s10461-10010-19711-10462. (2010).

124 Solomon, S. S. *et al.* High HIV prevalence and incidence among MSM across 12 cities in India. *AIDS.* **29**, 723-731. doi: 710.1097/QAD.0000000000000602. (2015).

125 Uma, S. *et al.* Bacterial vaginosis in female sex workers in Chennai, India. *Sex Health* **2**, 261-262, doi:10.1071/sh05025 (2005).

126 Venkatesh, K. K. *et al.* Sexual risk behaviors among HIV-infected South Indian couples in the HAART era: implications for reproductive health and HIV care delivery. *AIDS Care* **23**, 722-733, doi:10.1080/09540121.2010.525616 (2011).

127 Becker, M. *et al.* Etiology and determinants of sexually transmitted infections in Karnataka state, south India. *Sex Transm Dis* **37**, 159-164, doi:10.1097/OLQ.0b013e3181bd1007 (2010).

128 Patwardhan, V. & Bhalla, P. Role of type-specific herpes simplex virus-1 and 2 serology as a diagnostic modality in patients with clinically suspected genital herpes: A comparative study in Indian population from a tertiary care hospital. *Indian J Pathol Microbiol.* **59**, 318-321. doi: 310.4103/0377-4929.188104. (2016).

129 Shivanand, D. R., Murthy, S. C. & Raghu, T. Y. Genital herpes: A clinical and seroepidemiological study of patients attending a sexually transmitted diseases clinic in South India. *Journal of Pakistan Association of Dermatologists* **29**, 309-315 (2019).

130 Davies, S. C. *et al.* Prevalence and risk factors for herpes simplex virus type 2 antibodies among low- and high-risk populations in Indonesia. *Sex Transm Dis.* **34**, 132-138. (2007).

131 Joesoef, M. R. *et al.* Douching and sexually transmitted diseases in pregnant women in Surabaya, Indonesia. *J Formos Med Assoc.* **95**, 13-18. (1996).

132 Keorochana, N., Intaraprasong, W. & Choontanom, R. Herpesviridae prevalence in aqueous humor using PCR. *Clinical Ophthalmology* **12**, 1707-1711, doi:<http://0-dx.doi.org.elibrary.qatar-weill.cornell.edu/10.2147/OPTH.S174694> (2018).

133 Le, H. V. *et al.* Herpes simplex virus type-2 seropositivity among ever married women in South and north Vietnam: a population-based study. *Sex Transm Dis.* **36**, 616-620. doi: 610.1097/OLQ.1090b1013e3181a1098cde1094. (2009).

134 Ngo, T. D. *et al.* Use of commercial enzyme immunoassays to detect antibodies to the herpes simplex virus type 2 glycoprotein G in a low-risk population in Hanoi, Vietnam. *Clin Vaccine Immunol.* **15**, 382-384. Epub 2007 Dec 2012. (2008b).

135 Smith, J. S. *et al.* Prevalence and risk factors for herpes simplex virus type 2 infection among middle-age women in Brazil and the Philippines. *Posit Health News.*, 18. (2001).

136 Smith, J. S. *et al.* Herpes simplex virus-2 as a human papillomavirus cofactor in the etiology of invasive cervical cancer. *Journal of the National Cancer Institute* **94**, 1604-1613 (2002).

137 Sukvirach, S. *et al.* Population-based human papillomavirus prevalence in Lampang and Songkla, Thailand. *The Journal of infectious diseases* **187**, 1246-1256 (2003).

138 van de Wijgert, J. H., Kilmarx, P. H., Jones, H. E., Karon, J. M. & Chaikummao, S. Differentiating normal from abnormal rates of genital epithelial findings in vaginal microbicide trials. *Contraception.* **77**, 122-129. doi: 110.1016/j.contraception.2007.1010.1006. Epub 2007 Dec 1027. (2008).

139 Celentano, D. D. *et al.* Sexually transmitted infections and sexual and substance use correlates among young adults in Chiang Mai, Thailand. *Sex Transm Dis* **35**, 400-405, doi:10.1097/OLQ.0b013e31815fd412 (2008).

140 Dobbins, J. G. *et al.* Herpes in the time of AIDS: a comparison of the epidemiology of HIV-1 and HSV-2 in young men in northern Thailand. *J Oral Pathol Med.* **28**, 122-127. (1999).

141 Go, V. F. *et al.* High HIV sexual risk behaviors and sexually transmitted disease prevalence among injection drug users in Northern Vietnam: implications for a generalized HIV epidemic. *J Acquir Immune Defic Syndr.* **42**, 108-115. (2006).

142 Nguyen, N. T., Nguyen, H. T., Trinh, H. Q., Mills, S. J. & Detels, R. Clients of female sex workers as a bridging population in Vietnam. *AIDS Behav.* **13**, 881-891. doi: 810.1007/s10461-10008-19463-10464. Epub 12008 Oct 10462. (2009).

143 Pisani, E. *et al.* Basing policy on evidence: Low HIV, STIs, and risk behaviour in Dili, East Timor argue for more focused interventions. *Sex Transm Infect* **82**, 88-93, doi:<http://0-dx.doi.org.elibrary.qatar-weill.cornell.edu/10.1136/sti.2005.015602> (2006).

144 Limpakarnjanarat, K. *et al.* HIV-1 and other sexually transmitted infections in a cohort of female sex workers in Chiang Rai, Thailand. *Sex Transm Infect* **75**, 30-35 (1999).

145 Theng, T. S., Sen, P. R., Tan, H. H., Wong, M. L. & Chan, K. W. Seroprevalence of HSV-1 and 2 among sex workers attending a sexually transmitted infection clinic in Singapore. *Int J STD AIDS.* **17**, 395-399. (2006b).

146 van Griensven, F. *et al.* Evidence of an explosive epidemic of HIV infection in a cohort of men who have sex with men in Thailand. *AIDS* **27**, 825-832, doi:10.1097/QAD.0b013e32835c546e (2013).

147 Vu Thuong, N. *et al.* Impact of a community sexually transmitted infection/HIV intervention project on female sex workers in five border provinces of Vietnam. *Sex Transm Infect* **83**, 376-382, doi:10.1136/sti.2006.022616 (2007).

148 Chu, K. *et al.* Association between HSV-2 and HIV-1 viral load in semen, cervico-vaginal secretions and genital ulcers of Thai men and women. *Int J Std Aids* **17**, 681-686, doi:Doi 10.1258/095646206780071108 (2006).

149 Yap, S. H. *et al.* HIV/Human herpesvirus co-infections: Impact on tryptophan-kynurenine pathway and immune reconstitution. *PLoS One.* **12**, e0186000. doi: 0186010.0181371/journal.pone.0186000. eCollection 0182017. (2017).

150 Theng, C. T. *et al.* Seroprevalence of herpes simplex virus-1 and -2 in attendees of a sexually transmitted infection clinic in Singapore. *Sex Health.* **3**, 269-274. (2006a).

151 Rezza, G. *et al.* Human herpesvirus-8 and other viral infections, Papua New Guinea. *Emerging Infectious Diseases* **7**, 893 (2001).

152 Suligoi, B. *et al.* Infection with human immunodeficiency virus, herpes simplex virus type 2, and human herpes virus 8 in remote villages of southwestern Papua New Guinea. *Am J Trop Med Hyg.* **72**, 33-36. (2005).

153 Vallely, L. M. *et al.* Prevalence and risk factors of Chlamydia trachomatis, Neisseria gonorrhoeae, Trichomonas vaginalis and other sexually transmissible infections among women attending antenatal clinics in three provinces in Papua New Guinea: a cross-sectional survey. *Sex Health* **2** (2016).

154 Vallely, A. J. *et al.* Dorsal longitudinal foreskin cut is associated with reduced risk of HIV, syphilis and genital herpes in men: a cross-sectional study in Papua New Guinea. *J Int AIDS Soc.* **20**, 21358. doi: 21310.27448/IAS.21320.21301/21358. (2017).

155 Ryan, C. E. *et al.* Comparative performance of the Kalon and HerpeSelect enzyme-linked immunosorbant assays to determine the prevalence of herpes simplex virus type 2 in Papua New Guinea. *Sex Health.* **11**, 575-579. doi: 510.1071/SH14055. (2014).

156 Brijwal, M. *et al.* Herpes Simplex Virus Type 1 Genital Ulcer Disease at a Tertiary Care Hospital in North India. *Clinical infectious diseases : an official publication of the Infectious Diseases Society of America* **68**, 1783–1784, doi:10.1093/cid/ciy943 (2019).

157 Chua, S. H. & Cheong, W. K. Genital ulcer disease in patients attending a public sexually transmitted disease clinic in Singapore: an epidemiologic study. *Ann Acad Med Singapore* **24**, 510-514 (1995).

158 Hooi, P. S., Chua, B. H., Karunakaran, R., Lam, S. K. & Chua, K. B. A retrospective review of mucocutaneous infections by human herpesvirus 1 and 2 in an urban population in Malaysia. *The Medical journal of Malaysia* **57**, 80-87 (2002).

159 Rajan, V., Doraisingham, M., Sng, E. & Lim, A. Polymicrobial aetiology of genital ulcers. *Singapore medical journal* **23**, 207-213 (1982).

160 Sen, P., Sun, Y. J., Tan, H. H., Tan, S. H. & Chan, R. Comparison of nested-polymerase chain reaction and virus culture for the diagnosis of genital herpes simplex virus infection. *Singapore Med J.* **49**, 466-469. (2008).

161 Thirumoorthy, T. *et al.* Purulent penile ulcers of patients in Singapore. *Zhonghua Zhong Liu Za Zhi.* **8**, 444-446. (1986).

162 Zainah, S., Cheong, Y. M., Sinniah, M., Gan, A. T. & Akbal, K. A microbiological study of genital ulcers in Kuala Lumpur. *Shi Yan Sheng Wu Xue Bao.* **24**, 307-315. (1991).

163 Bhattarakosol, P., Visaprom, S., Sangdara, A. & Mungmee, V. Increase of genital HSV-1 and mixed HSV-1 and HSV-2 infection in Bangkok, Thailand. *J Med Assoc Thai* **88 Suppl 4**, S300-304 (2005).

164 Cheong, W. K., Thirumoorthy, T., Doraisingham, S. & Ling, A. E. Clinical and laboratory study of first episode genital herpes in Singapore. *Int J Std Aids* **1**, 195-198, doi:10.1177/095646249000100309 (1990).

165 Chiam, C. W., Chan, Y. F. & Sam, I. C. Changing trends of genital herpes in Kuala Lumpur, Malaysia, 1982-2008. *Int J Std Aids* **21**, 450-451, doi:10.1258/ijsa.2010.009569 (2010).

166 Chio, M., Aminah, S., Osiecki, J., Lewinski, M. & Low, L. Performance Characteristics of an Automated Assay on the Cobas (R) 4800 System to Detect Herpes Simplex Virus from Genital Lesion Specimens with the Cobas (R) Hsv 1 and 2 Test. *Sex Transm Infect* **91**, A128-A129, doi:10.1136/sextrans-2015-052270.338 (2015).

167 Doraisingham, S., Thirumoorthy, T., Ling, A. E., Lee, C. T. & Lim, K. B. Genital herpes in Singapore. *Zhonghua Yi Xue Za Zhi.* **67**, 570-572. (1987).

168 Goto, T., Yamaguchi, Y., Hashido, M., Yoshikawa, H. & Kawana, T. Diagnosis of Genital Herpes by Polymerase Chain Reaction Amplification. *Microbiology and Immunology* **37**, 987-990, doi:10.1111/j.1348-0421.1993.tb01735.x (1993).

169 Ishiguro, T., Ozaki, Y., Matsunami, M. & Funakoshi, S. Clinical and virological features of herpes genitalis in Japanese women. *Comp Immunol Microbiol Infect Dis* **5**, 437-446. (1982).

170 Jacob, M., Rao, P. S., Sridharan, G. & John, T. J. Epidemiology & clinical profile of genital herpes. *J Nutr.* **119**, 294-298. (1989).

171 Kaneko, H., Iida, T., Aoki, K., Ohno, S. & Suzutani, T. Sensitive and rapid detection of herpes simplex virus and varicella-zoster virus DNA by loop-mediated isothermal amplification. *Journal of Clinical Microbiology* **43**, 3290-3296, doi:<http://0-dx.doi.org.elibrary.qatar-weill.cornell.edu/10.1128/JCM.43.7.3290-3296.2005> (2005).

172 Kao, C. L., Lee, C. N., Lee, W. L., Hsieh, M. T. & Shih, H. M. Isolation and typing of herpes simplex virus from clinical specimens collected at National Taiwan University Hospital, 1981-1990. *Genitourin Med.* **67**, 353-354. (1991).

173 Kawana, T. *et al.* Clinical and virologic studies on female genital herpes. *Obstetrics and gynecology* **60**, 456-461 (1982).

174 Mathew, R. *et al.* Herpes simplex virus 1 and 2 in herpes genitalis: A polymerase chain reaction-based study from Kerala. *Indian Journal of Dermatology* **63**, 475-478 (2018).

175 Theng, T. S. C. & Chan, R. K. W. Genital herpes in a sexually-transmitted infection clinic in Singapore: A 1-year retrospective study. *Annals of the Academy of Medicine Singapore* **33**, 200-203 (2004).
