# Supplementary material for: Epidemiology of herpes simplex virus type 2 in Asia: A systematic review, meta-analysis, and meta-regression
Source: Lancet Reg Health West Pac. 2021 Jun 9;12:100176. doi: 10.1016/j.lanwpc.2021.100176 (PMC8356094; doi:10.1016/j.lanwpc.2021.100176)
Supplement: Supplementary file 2 [file mmc2.docx]

Supplementary Table S1. Preferred Reporting Items for Systematic Reviews and Meta-analyses (PRISMA) checklist.

Supplementary Table S2. Data sources and search criteria for systematically reviewing HSV-2 epidemiology in Asia.

Supplementary Box S1. The 26 countries/territories included in our definition of Asia.

Supplementary Box S2. Variables extracted from relevant reports meeting the inclusion criteria.

Supplementary Box S3. Definitions of population type classifications.

Supplementary Box S4. Factors (variables) selected a priori and included in univariable and multivariable meta-regression analyses.

Supplementary Table S3. Studies reporting HSV-2 seroincidence in Asia.

Supplementary Table S4. Studies reporting HSV-2 seroprevalence in East Asia.

Supplementary Table S5. Studies reporting HSV-2 seroprevalence in South Asia.

Supplementary Table S6. Studies reporting HSV-2 seroprevalence in Southeast Asia among different populations.

Supplementary Table S7. Studies reporting HSV-2 seroprevalence in Papua New Guinea.

Supplementary Figure S1. Forest plots presenting outcomes of the pooled mean HSV-2 seroprevalence among different populations in Asia.

A) General populations

B) Intermediate-risk populations

C) Higher-risk populations

D) STI clinic attendees and symptomatic populations

E) HIV-positive individuals and individuals in HIV-discordant couples

F) Other populations

Supplementary Table S8. Univariable and multivariable meta-regression analyses for HSV-2 seroprevalence in Asia using the year of publication instead of the year of data collection as the time variable.

Supplementary Table S9. Studies reporting proportions of HSV-2 virus isolation in clinically diagnosed genital ulcer disease and in clinically diagnosed genital herpes in Asia.

Supplementary Figure S2. Forest plots presenting outcomes of the pooled mean proportions of HSV-2 virus isolation in clinically diagnosed genital ulcer disease and in clinically diagnosed genital herpes in Asia.

A) Patients with genital ulcer disease

B) Patients with genital herpes

Supplementary Table S10. Summary of precision assessment and risk of bias (ROB) assessment for studies reporting HSV-2 seroprevalence in Asia.

References
